# Electrolyte selection toward efficient photoelectrochemical glycerol oxidation on BiVO_4_†

**DOI:** 10.1039/d4sc01651c

**Published:** 2024-05-31

**Authors:** Heejung Kong, Siddharth Gupta, Andrés F. Pérez-Torres, Christian Höhn, Peter Bogdanoff, Matthew T. Mayer, Roel van de Krol, Marco Favaro, Fatwa F. Abdi

**Affiliations:** a Helmholtz-Zentrum Berlin für Materialien und Energie GmbH Hahn-Meitner-Platz 1 14109 Berlin Germany marco.favaro@helmholtz-berlin.de fatwa.abdi@helmholtz-berlin.de; b Institut für Chemie & Biochemie, Freie Universität Berlin 14195 Berlin Germany; c Institut für Chemie, Technische Universität Berlin Straße des 17. Juni 124 10623 Berlin Germany; d School of Energy and Environment, City University of Hong Kong 83 Tat Chee Avenue Kowloon Hong Kong S.A.R. China ffabdi@cityu.edu.hk

## Abstract

Glycerol, a primary by-product of biodiesel production, can be oxidized into various value-added chemicals, significantly enhancing the techno-economic value of photoelectrochemical (PEC) cells. Several studies have explored various photoelectrode materials and co-catalysts, but the influence of electrolytes on PEC glycerol oxidation has remained relatively unexplored despite its significance. Here, we explore the impact of various acidic (pH = 2) electrolytes, namely NaNO_3_, NaClO_4_, Na_2_SO_4_, K_2_SO_4_, and KP_i_, on PEC glycerol oxidation using nanoporous thin film BiVO_4_ as a model photoanode. Our experimental findings reveal that the choice of electrolyte anion and cation significantly affects the PEC performance (*i.e.*, photocurrent, onset potential, stability, and selectivity towards value-added products) of BiVO_4_ for glycerol oxidation. To explain this interesting phenomenon, we correlate the observed performance trend with the ion specificity in the Hofmeister series as well as the buffering capacity of the electrolytes. Notably, NaNO_3_ is identified as the optimal electrolyte for PEC glycerol oxidation with BiVO_4_ when considering various factors such as stability and production rates for glycerol oxidation reaction (GOR) products, surpassing the previously favored Na_2_SO_4_. Glycolaldehyde emerges as the most dominant product with ∼50% selectivity in NaNO_3_. The general applicability of our findings is confirmed by similar observation in electrochemical (EC) GOR with a polycrystalline platinum anode. Overall, these results emphasize the critical role of electrolyte selection in enhancing the efficiency of EC/PEC glycerol oxidation.

## Abstract 2

Glycerol, a primary by-product of biodiesel production, can be oxidized into various value-added chemicals, significantly enhancing the techno-economic value of photoelectrochemical (PEC) cells.

## Graphical abstract

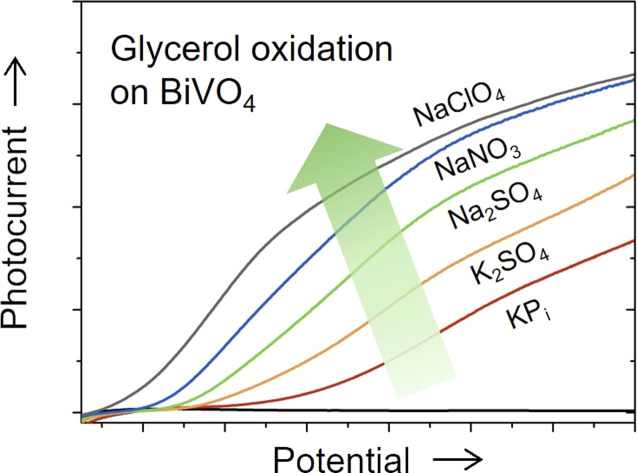

## Introduction

Glycerol, a major by-product of biodiesel production, holds significant potential as a feedstock to produce value-added chemicals.^1^ A broad palette of high-value products from the glycerol oxidation reaction (GOR), including dihydroxyacetone (DHA), formic acid (FA), glyceraldehyde (GLAD), and glycolaldehyde (GCAD), can be obtained in one or more reaction steps.^2^ Glycerol can also be oxidized electrochemically, which means it can serve as an anodic reactant in electrochemical (EC) or photoelectrochemical (PEC) devices. The oxidation of glycerol to *e.g.*, DHA in an aqueous environment can be written asCH_2_OH − CHOH − CH_2_OH + 2OH^−^ → CH_2_OH − CO − CH_2_OH + 2H_2_O + 2e^−^

and the corresponding reduction reaction as2H_2_O + 2e^−^ → H_2_ + 2OH^−^

For acidic environments, the reactions are written asCH_2_OH − CHOH − CH_2_OH → CH_2_OH − CO − CH_2_OH + 2H^+^ + 2e^−^2H^+^ + 2e^−^ → H_2_

The overall reaction is given asCH_2_OH − CHOH − CH_2_OH → CH_2_OH − CO − CH_2_OH + H_2_

The oxidation of glycerol (Δ*G* = 3.9 kJ mol^−1^, when coupled with the hydrogen evolution reaction, HER) requires significantly less energy and can be driven at much lower overpotentials compared to hydrogen production *via* the direct water splitting reaction (Δ*G* = 237.2 kJ mol^−1^, requiring an additional overpotential of at least 0.3 V for the oxygen evolution reaction, OER). Moreover, GOR products possess significantly higher economic value than glycerol. For instance, the market price of DHA ranges from approximately 2 to 150 € kg^−1^, depending on the degree of purity.^3^ In contrast, refined glycerol (≥99%) is available for ∼0.75 € kg^−1^, while the worldwide wholesale price for crude glycerol (∼80% purity) was ∼0.1 € kg^−1^ in 2020.^4^ Therefore, the techno-economic case for this PEC Power-to-X process is much more attractive than that for PEC water splitting.

In many (photo)electrochemical reactions, the composition of the electrolyte is known to be crucial.^5^ For example, Ding *et al.* showed that the choice of cations in alkaline electrolytes affects the water oxidation performance of TiO_2_ photoanodes. This was attributed to different back-reaction rates (*i.e.*, oxygen reduction reaction) induced by the various cations.^6^ Similarly, the choice of anions has been shown to influence the performance of WO_3_ photoanodes, as these anions can be parasitically oxidized alongside water at the electrode surface.^5*a*,7^ In the case of PEC glycerol oxidation, too few studies are available to determine the optimal supporting electrolyte for achieving high efficiency and stability. Since Liu *et al.* demonstrated that glycerol oxidation using BiVO_4_ photoanodes exhibited the highest efficiency in acidic solutions (pH = 2) based on sodium sulfate (Na_2_SO_4_),^8^ subsequent studies mainly used Na_2_SO_4_-based electrolytes and predominantly focused on modifying the photoanode material and/or the deposited co-catalyst to optimize the performance.^9^ It is still unclear whether altering the electrolyte composition could also optimize the PEC glycerol oxidation performance.

In this paper, we demonstrate that the electrolyte composition substantially impacts the glycerol oxidation performance of our model photoanode material, bismuth vanadate (BiVO_4_). We investigate acidic electrolytes (pH = 2) with different cations and anions: sodium nitrate (NaNO_3_), sodium perchlorate (NaClO_4_), sodium sulfate (Na_2_SO_4_), potassium sulfate (K_2_SO_4_), and potassium phosphate (KP_i_), the latter being a pH buffer solution. Our systematic study reveals that, when performance factors such as photocurrent, stability, and production rates towards high-value GOR products are comprehensively considered, BiVO_4_ photoanodes exhibit the highest glycerol oxidation performance in NaNO_3_, surpassing the commonly employed Na_2_SO_4_. To the best of our knowledge, NaNO_3_ has not been previously reported as an electrolyte for PEC GOR. The underlying reason behind this observed performance difference is discussed, particularly in relation to the classification of the ions in the Hofmeister series and the pH buffering capacities of the employed solutions. Furthermore, the same GOR performance trend with the electrolyte choice is also observed when the BiVO_4_ photoanode is replaced with a polycrystalline platinum (Pt) anode, suggesting that our explanations may be broadly applicable, irrespective of the anode material or whether the process is EC- or PEC-driven. Overall, these findings emphasize the critical role of electrolyte selection for achieving high performance and stability in glycerol oxidation and underscore the potential of NaNO_3_ as a favorable electrolyte for glycerol oxidation.

## Results and discussion

BiVO_4_ photoanodes were prepared using a previously reported electrodeposition method,^10^ and the detailed synthesis method is described in the ESI.† The samples exhibit a nanoporous morphology (Fig. S1†), while the X-ray diffractogram shows a monoclinic crystalline phase without any impurities (Fig. S2a†). Tauc analysis of the samples revealed an indirect bandgap of 2.48 eV (Fig. S2b†), which is within the range of typical bandgap values reported for monoclinic BiVO_4_.^11^

**Fig. 1:**
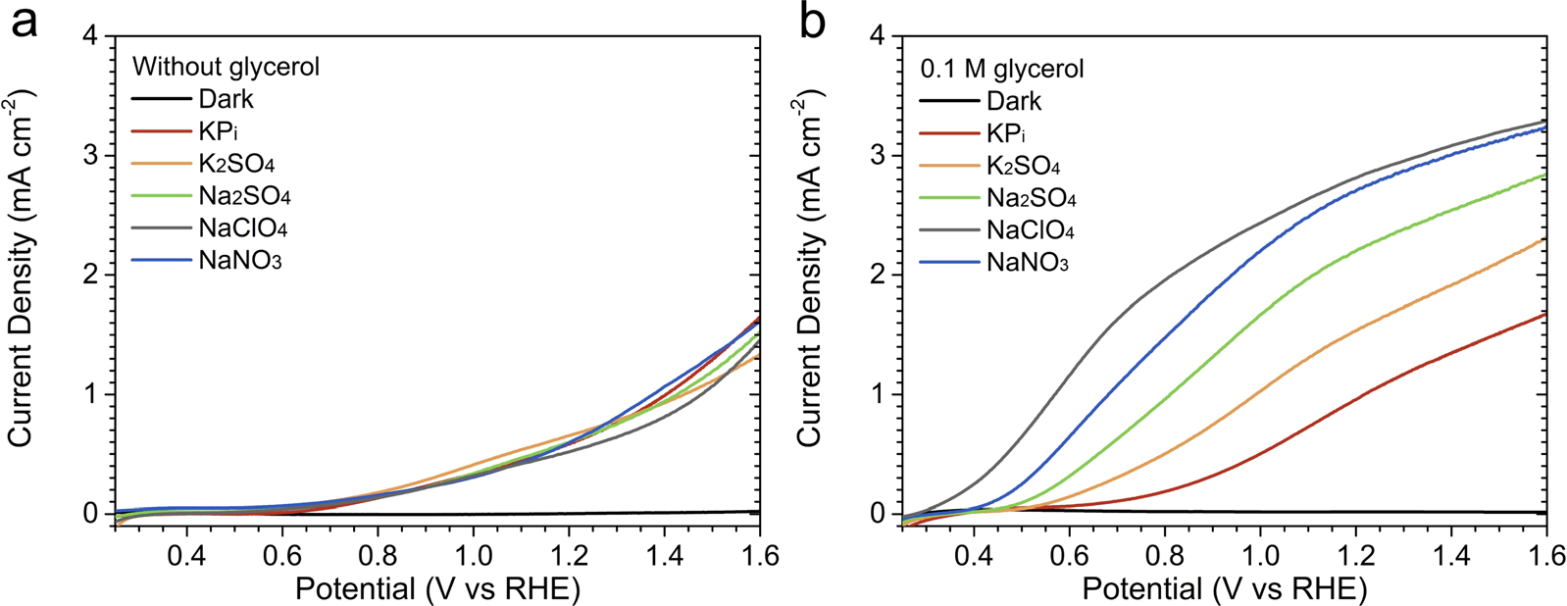
Linear sweep voltammetry (LSV) curves recorded in different acidic solutions (pH = 2), all having a concentration of 0.5 M: (a) in the absence of glycerol and (b) with 0.1 M glycerol. Measurements were performed at a scan rate of 20 mV s^−1^, sweeping from negative to positive potentials relative to the reversible hydrogen electrode (RHE), under AM 1.5G simulated sunlight illumination. LSV curves recorded in NaNO_3_ without illumination are also shown as the representative dark curves; dark LSV curves recorded in other electrolytes are shown in Fig. S3.†

We first compare the PEC performance of BiVO_4_ toward water oxidation in various acidic electrolytes, in the absence of glycerol. Fig. 1a shows the linear sweep voltammetry (LSV) curves measured under AM 1.5G simulated sunlight (100 mW cm^−2^) in KP_i_, K_2_SO_4_, Na_2_SO_4_, NaClO_4_, and NaNO_3_ (see Fig. S3a† for the corresponding dark LSV curves). The concentration of all electrolytes was kept constant at 0.5 M, and the pH value was adjusted to 2. To avoid altering the ionicity of the solutions, the pH of each investigated electrolyte was lowered using mineral acids with the same anion of the electrolyte itself (*e.g.*, in the case of NaNO_3_, the pH was lowered using HNO_3_, whereas H_2_SO_4_ was used for K_2_SO_4_). Our electrodeposited BiVO_4_ photoanodes showed similar photocurrent (0.5–0.7 mA cm^−2^) at 1.23 V *vs.* the reversible hydrogen electrode (*V*_RHE_) and onset potential (∼0.6 V_RHE_) in all electrolytes (see Fig. S4 and Table S1† for the measurements' reproducibility), indicating that the difference in the electrolyte composition does not affect their performance toward water oxidation.

To investigate the effect of electrolyte composition on the performance of PEC glycerol oxidation using BiVO_4_, 0.1 M glycerol was added to each of the five electrolyte solutions. Fig. 1b displays the LSV curves measured under illumination (also see Fig. S5 and Table S2† for reproducibility), while the LSV curves obtained in the absence of illumination are presented in Fig. S3b.† In the presence of 0.1 M glycerol, our BiVO_4_ photoanodes showed increased photocurrent in all electrolytes. This is attributed to the lower overpotential needed for the GOR as compared to the OER. Interestingly, the photocurrents varied significantly depending on the electrolyte composition. The lowest photocurrent was obtained in the KP_i_ solution, followed by the K_2_SO_4_, Na_2_SO_4_, NaNO_3_, and NaClO_4_ solutions. The photocurrent improvement was rather significant, with 180% increase by changing the electrolyte from KP_i_ to NaClO_4_ (1.07 ± 0.08 *vs.* 2.97 ± 0.11 mA cm^−2^ at 1.23 V_RHE_).

The same photocurrent trend was also observed when the glycerol concentration was increased. Fig. S6† shows the LSV curves of BiVO_4_ photoanodes measured with 0.5 M glycerol. As in the case of 0.1 M glycerol, our photoanodes showed the lowest photocurrent in the KP_i_ solution and the highest in the NaClO_4_ solution. However, the photocurrents in the NaNO_3_ and NaClO_4_ solutions were similar, especially at higher potentials (>1.0 V_RHE_). The photocurrent difference was more pronounced at relatively low potentials. For example, in the NaNO_3_ and NaClO_4_ solutions, the photocurrent already reached 1.0 mA cm^−2^ at approximately 0.5 V_RHE_, whereas in the KP_i_ solution, a potential of 0.9 V_RHE_ was needed.

**Fig. 2:**
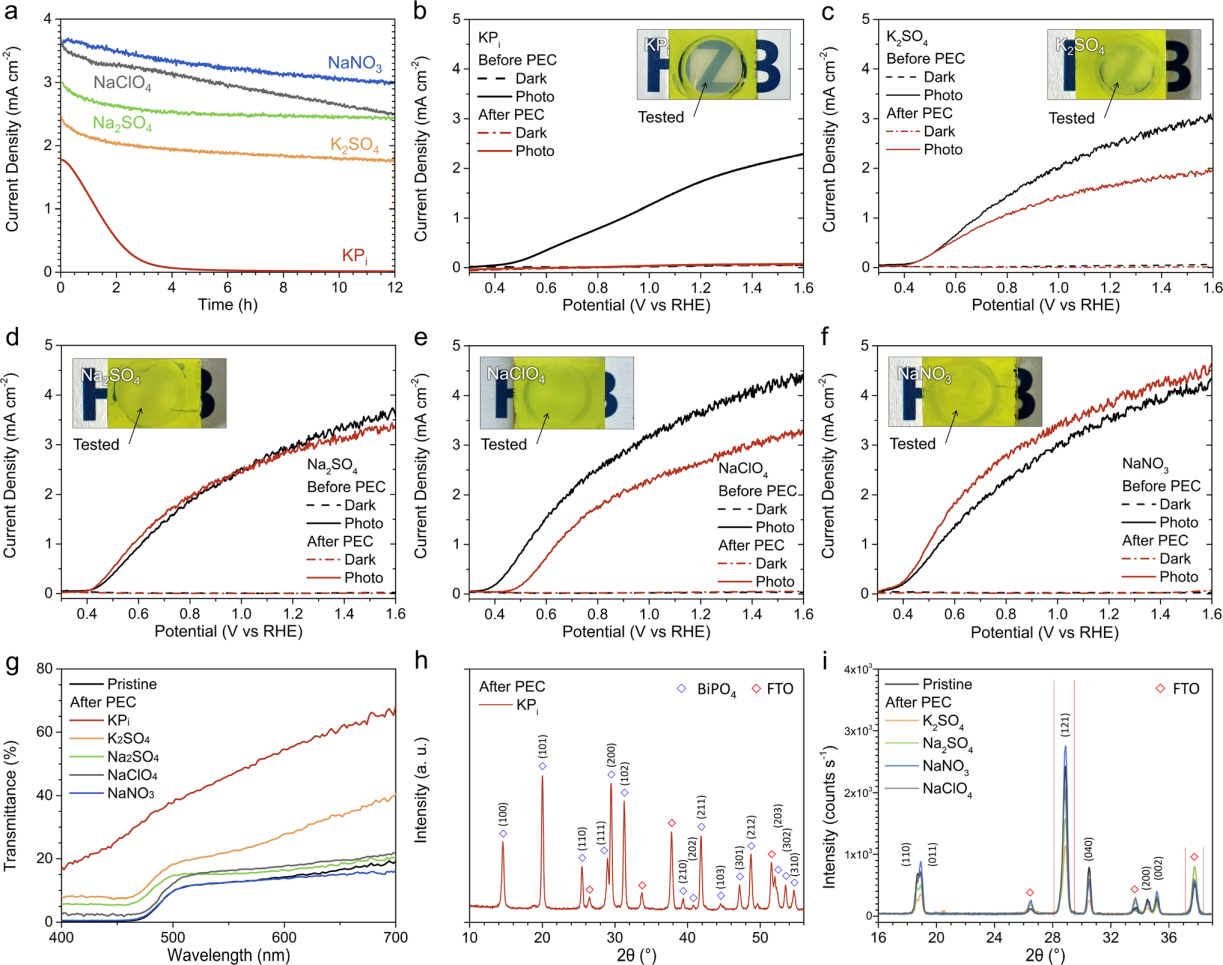
(a) Chronoamperometry (CA) curves measured at 1.23 V_RHE_ under AM 1.5G illumination in different acidic electrolyte solutions (pH = 2) containing 0.5 M glycerol. Linear sweep voltammetry (LSV) curves measured under AM 1.5G illumination before (black curves) and after (red curves) the 12 hours CA measurements in (b) KP_i_, (c) K_2_SO_4_, (d) Na_2_SO_4_, (e) NaClO_4_, and (f) NaNO_3_ solutions containing 0.5 M glycerol. Insets in (b)–(f) show the digital photographs of the specimens after undergoing the 12 hours CA tests in the different acidic electrolytes in the presence of 0.5 M glycerol. The circular areas exposed to the electrolyte during the tests are indicated. (g) UV-vis transmittance of pristine BiVO_4_ and BiVO_4_ samples following the 12 hours CA measurements shown in (a). X-ray diffractogram of the BiVO_4_ samples after the CA tests (h) in KP_i_ solution and (i) in K_2_SO_4_, Na_2_SO_4_, NaClO_4_, and NaNO_3_ solutions.

Chronoamperometry (CA) was performed at 1.23 V_RHE_ for a duration of 12 hours to assess the stability of our photoanodes in the five acidic electrolytes containing 0.5 M glycerol, as reported in Fig. 2a. In the KP_i_ solution, the photocurrent decreased rapidly to 1/*e* (36.7%) of its initial value within only 1.8 hours, ultimately resulting in a complete loss of photocurrent within 8 hours. In contrast, the photoanodes exhibited photocurrent retention of approximately 70–80% over the 12 hours period in K_2_SO_4_, Na_2_SO_4_, NaClO_4_, and NaNO_3_. The corresponding LSV curves obtained before and after the CA are shown in Fig. 2b–f. After the CA measurement in KP_i_, no photoactivity remained (see Fig. 2b), consistent with the complete photocurrent loss during the CA experiment. The samples tested in K_2_SO_4_ and NaClO_4_ also showed a consistent photocurrent decrease after the CA (see Fig. 2c and e). Interestingly, the samples tested in Na_2_SO_4_ and NaNO_3_ maintained similar photocurrent levels in their LSV measurements compared to those observed before the 12 hours CA, indicating some degree of recovery (see Fig. 2d and f). Note that we limit our study to pH 2 electrolyte solutions, as the use of more acidic pH (*e.g.*, pH 1) renders the BiVO_4_ to be unstable even in the NaNO_3_ solution (Fig. S7†).

The photographs of the sample taken after the 12 hours CA in each electrolyte solution are presented in the insets of Fig. 2b–f. In the case of the samples tested in KP_i_, the exposed area became white and transparent. This is not the case for samples tested in K_2_SO_4_, Na_2_SO_4_, NaClO_4_, and NaNO_3_; no significant difference was observed between the appearance of the tested and untested regions. We quantified our observation by measuring the UV-vis transmittance of these regions, as shown in Fig. 2g. Notably, the transmittance of the sample tested in NaNO_3_ closely matched that of the pristine sample across the entire wavelength range. The samples tested in K_2_SO_4_, Na_2_SO_4_, and NaClO_4_ exhibited a notable increase in transmittance, albeit with varying amount, suggesting the possible loss of BiVO_4_ material due to photocorrosion and consequent dissolution. The sample tested in KP_i_ displayed significantly higher transmittance across the entire wavelength range, consistent with the appearance of the sample after electrolysis (see inset of Fig. 2b).

We then compared the crystallinity of the BiVO_4_ samples before and after the 12 hours CA measurements. Fig. 2h displays the X-ray diffractogram for the sample tested in KP_i_. Diffraction peaks corresponding to BiPO_4_ (as well as the FTO substrate) were detected, while peaks characteristic of monoclinic BiVO_4_ were absent. This suggests that during the CA measurement, vanadium atoms in BiVO_4_ leached into the electrolyte and were substituted by phosphorus atoms from the KP_i_ solution, leading to a crystal structure transformation and eventually a complete conversion to BiPO_4_. Indeed, BiPO_4_ has a wide bandgap (∼3.85 eV),^12^ which explains the white and transparent appearance of the film after CA. Such a conversion could also account for the low photocurrent observed with KP_i_ (Fig. 1b). Note that the formation of a BiPO_4_ thin film on the surface of BiVO_4_ upon exposure to KP_i_ buffer solution at pH 7 has been previously reported.^13^ A full conversion to BiPO_4_ has not been reported, which we attribute to the use of lower pH.

Fig. 2i shows the X-ray diffractograms of the BiVO_4_ samples measured after the 12 hours CA measurements in K_2_SO_4_, Na_2_SO_4_, NaClO_4_, and NaNO_3_. Unlike the sample measured in KP_i_, diffraction peaks characteristic of monoclinic BiVO_4_ can be detected in these samples. For samples measured in K_2_SO_4_, Na_2_SO_4_, and NaClO_4_, the intensities of the (121) diffraction peak, which is the most prominent reflection for electrodeposited monoclinic BiVO_4_^10^, decreased, while the intensities of the FTO peak at 2*θ* = 37.6° increased. In the case of the sample measured in NaNO_3_, the X-ray diffraction (XRD) pattern closely resembled that of the pristine sample. We quantified the area under the (121) peak and the FTO peak at 2*θ* = 37.6° and then calculated their ratio. For the sample tested in NaNO_3_, this ratio was 4.8, which was higher than that of the pristine sample (3.9). In contrast, the ratios for the samples tested in NaClO_4_, Na_2_SO_4_, and K_2_SO_4_ were considerably lower; 2.8 for NaClO_4_, 1.8 for Na_2_SO_4_, and 1.4 for K_2_SO_4_, respectively.

The XRD and UV-vis transmittance results above indicate that BiVO_4_ substantially degraded during GOR in acidic NaClO_4_, Na_2_SO_4_ and K_2_SO_4_, likely due to photocorrosion, but not in NaNO_3_. These findings are interesting because, according to the Pourbaix diagram,^14^ BiVO_4_ is expected to decompose into BiO^+^ and VO_4_^3−^ at pH 2 and 1.23 V_RHE_. This suggests that NO_3_^−^ (either by itself or in conjunction with glycerol) plays a role in stabilizing BiVO_4_ under these conditions. The exact mechanism requires further investigation beyond the scope of this study.

The surface chemical characteristics of the pristine sample and samples subjected to 12 hours CA in glycerol-containing K_2_SO_4_, Na_2_SO_4_, NaClO_4_, and NaNO_3_ electrolytes were investigated using X-ray photoelectron spectroscopy (XPS). The sample subjected to the same treatment in KP_i_ was not tested since XRD already showed a clear transformation to BiPO_4_. The Bi 4f core-level spectra are displayed in Fig. S8,† and the O 1s and V 2p core-level spectra are presented in Fig. S9.† While the binding energy difference (ΔBE) between the Bi 4f_5/2_ and 4f_7/2_ peaks remained constant, the tested samples exhibited a slight shift towards higher binding energies. The magnitude of this peak shift was 0.1 eV in the NaNO_3_ solution and increased to 0.2 eV in the K_2_SO_4_, Na_2_SO_4_, and NaClO_4_ solutions. A notable difference among the samples was observed in the O 1s core-level spectra. The samples tested in the K_2_SO_4_, Na_2_SO_4_, and NaClO_4_ solutions showed distinct peaks at higher binding energies of approximately 531–533 eV. These peaks could originate from adsorbed hydroxyl ions (at ∼532 eV),^15^ likely due to glycerol adsorption during GOR. Their significant emergence may imply a somewhat reduced surface chemical stability of BiVO_4_ during PEC oxidation of glycerol in the K_2_SO_4_, Na_2_SO_4_, and NaClO_4_ solutions at pH 2. Indeed, if the O^2−^ ions in BiVO_4_ were substituted by hydroxyl (OH^−^) ions, the resulting decrease in the number of valence electrons could potentially lead to a shift to higher binding energies in the Bi 4f and V 2p peaks. In contrast, the sample tested in NaNO_3_ solution exhibited only a slight increase in signal intensity near 532 eV, indicating the relatively superior chemical stability of BiVO_4_ in pH 2 NaNO_3_ solution during PEC glycerol oxidation. Overall, these XPS findings are in agreement with the XRD and UV-vis results.

**Fig. 3:**
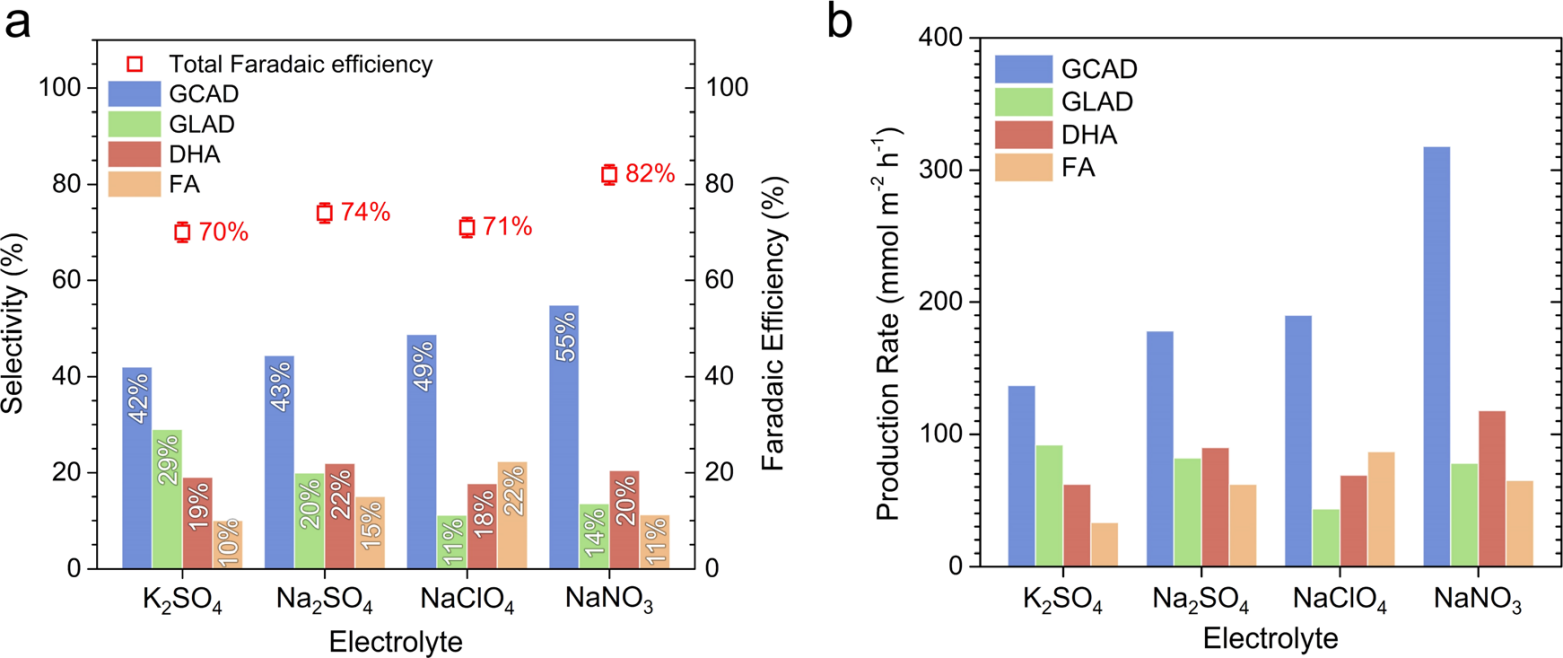
Product analysis using high-performance liquid chromatography (HPLC). (a) Selectivity and (b) production rate towards glycerol oxidation reaction (GOR) products in various acidic electrolytes (pH = 2). Total faradaic efficiency, calculated in terms of charges used for GOR, is presented on the right *y*-axis in (a). The methods for calculating selectivity, production rate, and faradaic efficiency are described in the ESI.† Liquid samples were collected after 12 hours photoelectrolysis at a constant potential of 1.23 V_RHE_, corresponding to the measurements shown in Fig. 2a. GCAD: glycolaldehyde, GLAD: glyceraldehyde, DHA: dihydroxyacetone, FA: formic acid.

The selectivity and production rates towards value-added GOR products during the 12 hours CA tests in various acidic electrolyte solutions were analyzed using high-performance liquid chromatography (HPLC). Detailed experimental methods for the HPLC analyses are provided in the ESI† (also refer to Fig. S10–S12† for calibration information). KP_i_ was excluded from our analysis due to the severe instability of BiVO_4_ during the GOR in the pH 2 KP_i_ solution (*vide supra*). Fig. 3a displays the selectivity of BiVO_4_ towards various GOR products and the total faradaic efficiency, calculated in terms of charges used for GOR, in K_2_SO_4_, Na_2_SO_4_, NaClO_4_, and NaNO_3_ solutions (the corresponding chromatograms are shown in Fig. S13–S16†). In our study, GCAD (C_2_H_4_O_2_), GLAD (C_3_H_6_O_3_), DHA (C_3_H_6_O_3_), and FA (CH_2_O_2_) were detected as the GOR products. Notably, GCAD emerged as the most dominant product in all electrolytes, which is consistent with a recent report.^16^ However, the selectivity varied depending on the electrolyte. In K_2_SO_4_, the selectivity for GLAD (28%) a C3 molecule, was the highest among the electrolytes tested. Furthermore, the selectivity for FA, the only C1 product in our case, was as low as 10%. Interestingly, in the Na_2_SO_4_, NaClO_4_, and NaNO_3_ solutions, the selectivities for GCAD (C2) and FA (C1) increased, while the selectivity for GLAD (C3) decreased (the selectivity for DHA remained relatively constant at ∼20% in all electrolytes). In NaNO_3_, the selectivity for GCAD exceeded 50% and the selectivity for GLAD was as low as 14%. These results suggest that the rate of C–C cleavage in GLAD, which was proposed as a pathway to the formation of GCAD and FA,^16^ might differ among various electrolyte solutions, consequently influencing the overall GOR performance. One possible explanation is that GLAD adsorbs more favorably onto the BiVO_4_ surface in the NaNO_3_ solution compared to others. Further computational and/or experimental studies beyond the scope of our work are needed to reveal the actual mechanism. Fig. 3b presents the production rates of the four GOR products. In the NaNO_3_ solution, the production rate of GCAD was calculated to be 318 mmol m^−2^ h^−1^, which is, to the best of our knowledge, the highest reported value for undoped BiVO_4_ without any co-catalysts.

The total faradaic efficiency was highest in the NaNO_3_ solution, as shown in Fig. 3a, where an estimated 82% of the photo-generated holes were used for oxidizing glycerol. Mass spectroscopy (MS) results (see Fig. S17†) obtained from the NaNO_3_ solution indicate that no O_2_ was generated above our detection limit. This suggests that the OER was effectively suppressed in the NaNO_3_ solution, likely owing to the relatively low energy requirement of the GOR. A slight increase in the mass signal corresponding to CO_2_ was observed, suggesting the potential for complete oxidation of glycerol to CO_2_. We tentatively attribute the remaining photo-generated holes (∼18%) to the generation of other glycerol oxidation products including CO_2_ (undetected with HPLC and MS) or to photocorrosion. The lower faradaic efficiencies observed in Na_2_SO_4_ (74%), NaClO_4_ (71%), and K_2_SO_4_ (72%) support the hypothesis of partial hole consumption due to photocorrosion, especially given the poorer photostability of BiVO_4_ in these solutions. We also confirm that nitrate reduction reaction (NRR), which could potentially occur at the counter electrode when NaNO_3_ is used as the electrolyte, was not detected in our experiments (see Fig. S18†).

In terms of photocurrent, stability, and selectivity to high-value GCAD, NaNO_3_ was identified as the most suitable electrolyte for PEC glycerol oxidation using BiVO_4_. To elucidate how the choice of electrolyte influences the GOR performance of BiVO_4_, it is first necessary to examine the electrical conductivities of the electrolyte solutions. The electrical conductivities of 0.5 M NaNO_3_, NaClO_4_, Na_2_SO_4_, K_2_SO_4_, and KP_i_ solutions (all at pH 2) were measured as 75.5 mS cm^−1^, 98.2 mS cm^−1^, 102.6 mS cm^−1^, 141.2 mS cm^−1^, and 100.0 mS cm^−1^, respectively, which do not correlate with the trends observed in GOR performance. Moreover, if the differing GOR performance observed in BiVO_4_ was due to differences in electrical conductivity among the electrolytes, then the water-splitting performance of BiVO_4_ should have varied accordingly.

**Fig. 4:**
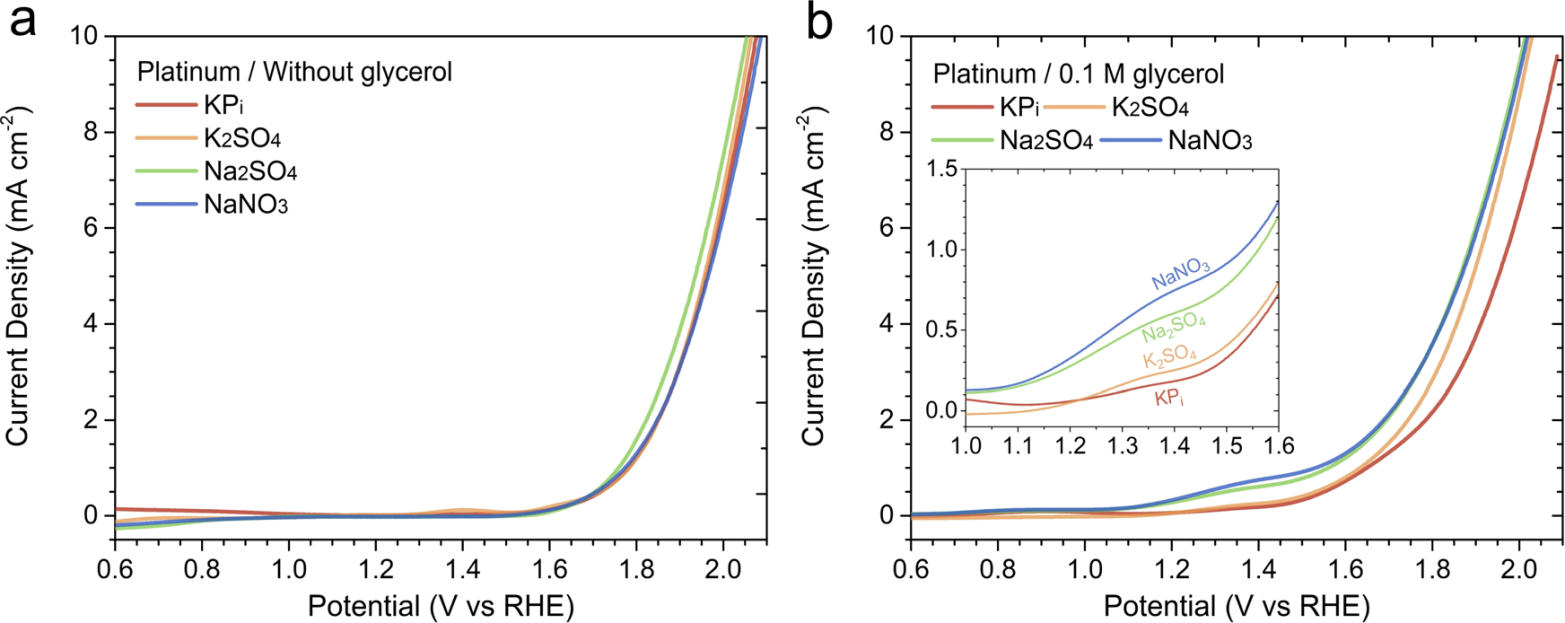
Linear sweep voltammetry (LSV) curves of a polycrystalline platinum (Pt) anode measured (a) without glycerol and (b) in the presence of 0.1 M glycerol in various acidic electrolytes at pH 2, with an electrolyte concentration of 0.5 M. Measurements were performed at a scan rate of 20 mV s^−1^.

Moreover, to determine whether the GOR performance trend in different acidic electrolytes is specific to BiVO_4_ and/or the PEC process, we examined the GOR performance of a polycrystalline Pt anode. Fig. 4a displays the LSV curves of the Pt anode measured without glycerol in various acidic electrolytes. Note that NaClO_4_ solution is excluded from this particular study, the formation of platinum oxide that degrades the activity has been reported when Pt is exposed to perchloric acid.^17^ The Pt anode exhibited similar LSV curves across all electrolytes. For example, the onset potential for OER was observed at approximately 1.6 V_RHE_, and an overpotential of ∼0.8 V was required to achieve 10 mA cm^−2^ (at ∼2.0 V_RHE_) in all solutions. Fig. 4b presents the LSV curves of the Pt anode measured in the presence of 0.1 M glycerol. The current density for the Pt anode began to increase earlier than in its absence, similar to the case with BiVO_4_, reflecting the lower energy requirement of GOR compared to OER. Interestingly, the Pt anode exhibited the same GOR performance trend as BiVO_4_ in terms of current density. At 1.4 V_RHE_, a potential more negative than the onset for OER, the current densities at 1.4 V_RHE_ ranked as follows: NaNO_3_ > Na_2_SO_4_ > K_2_SO_4_ > KP_i_ (see Fig. 4b). These results indicate that the influence of the electrolyte on GOR performance, particularly in terms of current density, is not unique to BiVO_4_ and may be generally applicable to other anode materials and whether the reaction is EC- or PEC-driven.

**Fig. 5:**
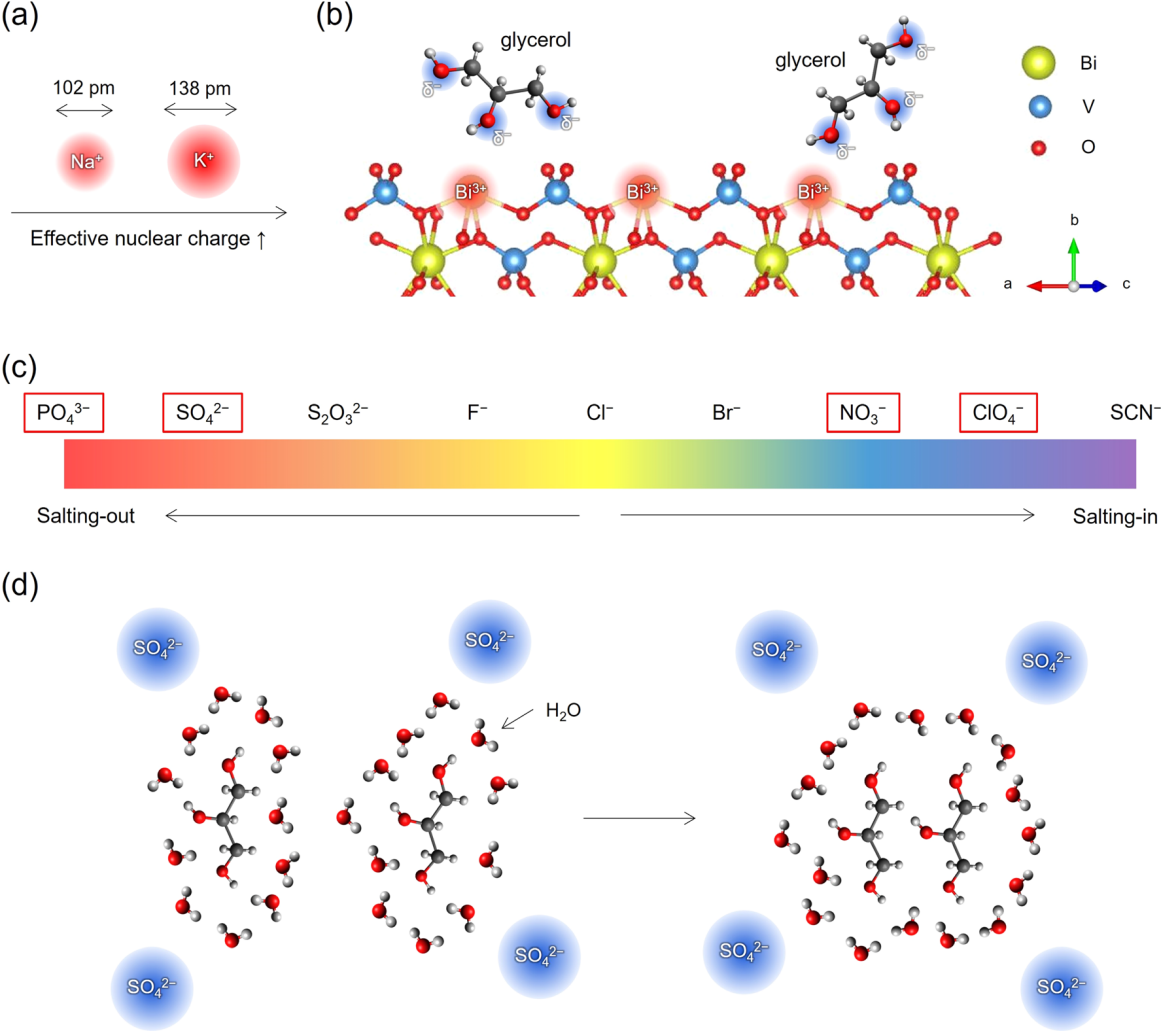
Schematic illustrations of (a) Na^+^ and K^+^ ions with distinct effective ionic radii, leading to varying effective nuclear charges, and (b) the BiVO_4_ surface interaction with glycerol: hydroxyls of glycerol, carrying a partial negative charge (*δ*^−^), are electrostatically drawn to the Bi^3+^ ions on the BiVO_4_ surface. (c) The Hofmeister series. (d) Schematic illustration of the aggregation of glycerol molecules induced by the presence of SO_4_^2−^ anions.

These findings lead us to investigate the impact of specific ions in the electrolytes on PEC glycerol oxidation performance, extending beyond mere electrolyte conductivity or anode material. Specifically, the photocurrent trend in the different electrolytes (for BiVO_4_: NaClO_4_ > NaNO_3_ > Na_2_SO_4_ > K_2_SO_4_ > KP_i_) suggests that the presence of specific ions in the electrolyte is beneficial/detrimental to the PEC glycerol oxidation performance. First, the trend indicates that Na^+^ is more favorable than K^+^ for GOR on BiVO_4_. We attribute this observation to the prominent difference between the cation size. As illustrated in Fig. 5a, K^+^ has an approximately 30% larger effective ionic radius of 138 pm compared to Na^+^ (102 pm),^18^ resulting in a larger effective nuclear charge than Na^+^.^19^ According to density functional theory (DFT) calculations performed by Liu *et al.*, the hydroxyl groups of glycerol spontaneously adsorb at the exposed Bi^3+^ sites of BiVO_4_ through electrostatic interactions between Bi^3+^ and oxygen in the hydroxyl groups, as depicted in Fig. 5b.^8^ Similarly, previous DFT calculations on glycerol adsorption to Pt surfaces also showed that the hydroxyl groups of glycerol adsorb to the surface Pt atoms. Furthermore, previous liquid chromatography-mass spectrometry results showed that glycerol molecules in Na_2_SO_4_ solutions (pH 2) were found in the form of C_3_H_8_O_3_Na (glycerol = C_3_H_8_O_3_).^8^ This observation indicates a strong interaction between the hydroxyl groups of glycerol, with their negative partial charge *δ*^−^, and the cations in the electrolytes. Given that K^+^ has a larger effective nuclear charge (defined as *Z*_eff_ = *Z* − *S*, where *Z* is the number of protons (atomic number) and *S* is the shielding constant),^20^ the glycerol hydroxyl groups are expected to interact more strongly with K^+^ than with Na^+^. Consequently, this can lead to stronger electrical neutralization of the hydroxyls of glycerol, which may weaken the electrostatically induced adsorption of glycerol molecules to the Bi^3+^ sites. As a result, GOR at BiVO_4_ in K^+^-containing electrolytes would be hindered.

In addition, the superior performance of BiVO_4_ in NaNO_3_ and NaClO_4_ than in Na_2_SO_4_ may be explained considering the different types of anions, NO_3_^−^, ClO_4_^−^, and SO_4_^2−^. The Pt anode also exhibited a higher GOR current in NaNO_3_ than in Na_2_SO_4_. Interestingly, we note that SO_4_^2−^ stands out as one of the anions exhibiting potent specific ion effects, positioned towards the left end of the Hofmeister series,^21^ as shown in Fig. 5c. This indicates a strong salting-out effect of SO_4_^2−^. In contrast, NO_3_^−^ and ClO_4_^−^ are positioned closer to the right end of the Hofmeister series, indicating that these anions exhibit comparatively weak specific ion effects and limited salting-out effects. Anions with relatively small sizes and high charge densities, such as SO_4_^2−^ and PO_4_^3−^, trigger the salting-out phenomenon in aqueous solutions by inducing electronic repulsion and enhancing the hydrophobic nature of the molecules involved.^22^ The stronger hydrophobicity prompts the molecules to aggregate, thereby minimizing the entropic cost related to a highly ordered structure at the interface between the solute and water, as illustrated in Fig. 5d. This aggregation of glycerol molecules could reduce the probability of glycerol adsorbing onto Bi^3+^ sites, thereby hindering GOR on BiVO_4_. It is noted that a similar anion effect following the trend of the Hofmeister series has also been recently discussed to explain different H_2_ microbubble coalescence efficiencies.^23^

**Fig. 6:**
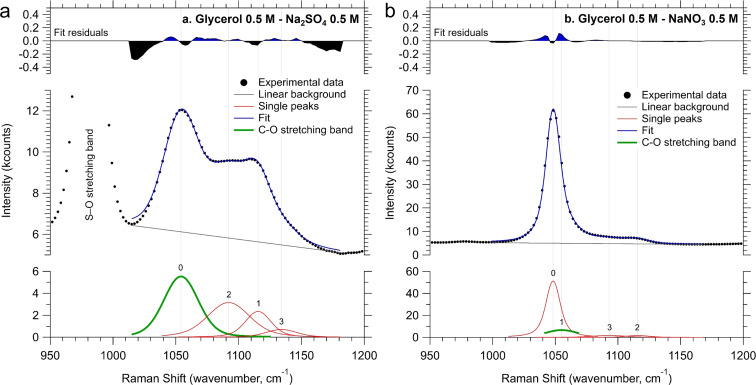
C–O stretching band region for the two Na-based electrolytes at pH 2, (a) Na_2_SO_4_ and (b) NaNO_3_, both containing 0.5 M glycerol. The spectra were recorded at approximately 22 °C, using a 2 mL optical grade-quartz cuvette, at a laser wavelength of 785 nm, and a total spectral power of 450 mW.

In order to experimentally investigate the aforementioned molecular aggregation, we performed Raman scattering spectroscopy on electrolyte solutions containing 0.5 M glycerol (the detailed method is described in the ESI†). We focused on the C–O stretching band, centered at around 1050 cm^−1^, as indicated by the grey area in the full range spectra reported in Fig. S19.†^24^ The band was scanned between 950 cm^−1^ and 1200 cm^−1^ and fitted with multiple symmetric Voigt functions after subtracting a linear background. Fig. S20a and S20b† report the fitting results for the spectral references, namely 0.5 M glycerol in water and 0.5 M of aqueous NaNO_3_. The latter was taken since a spectral overlap exists between the C–O and the N–O stretching bands. The line shape of the Voigt functions retrieved by fitting the spectral references was then kept constant during the fitting of the pristine electrolytes, with their spectral position and full-width at half-maximum (FWHM) allowed to adjust in a range of 5 cm^−1^ with respect to their values in the spectral references during the fitting procedure. Fig. 6a and b show the fitting procedure obtained on the 0.5 M Na_2_SO_4_ and NaNO_3_ solutions (pH 2) containing 0.5 M glycerol, respectively. Table 1 summarizes the Raman shift values of the C–O stretching band retrieved during the fitting procedure of the samples above and the corresponding difference with respect to the glycerol in water without any supporting electrolyte. It is evident that there is a red shift of the C–O stretching frequency, indicating that the reduced mass of the C–O stretching mode of glycerol increases upon the addition of salts. This phenomenon can be attributed to the formation of glycerol “aggregates” in the solution, driven by the coulombic interaction between the partial negative charge present on the oxygen of glycerol's OH groups and the ions introduced into the solution. Notably, the observed downward shift of the C–O stretching frequency is more pronounced with Na_2_SO_4_ than with NaNO_3_. These observations support our hypothesis that sulfate groups prompt the formation of slightly more glycerol aggregates in the solution.^25^

**Table 1:** Raman shift values of the C–O stretching band retrieved during the fitting procedure of the liquid samples, and the corresponding difference with respect to the glycerol in water without any supporting electrolyte

	C–O stretching Raman shift (cm^−1^)	C–O stretching ΔRaman shift (cm^−1^)
0.5 M glycerol	1055.8	—
0.5 M glycerol + 0.5 M NaNO_3_	1055.0	0.8
0.5 M glycerol + 0.5 M Na_2_SO_4_	1054.1	1.7

We also note that the photocurrent trend may also be explained by the ability of the different electrolytes in suppressing (or allowing) pH changes. When glycerol undergoes oxidation at the anode, protons are generated. These protons subsequently migrate to the cathode and participate in the HER. Since acidic environment is preferred for the GOR,^8^ the overall glycerol oxidation performance may be affected if protons produced near the BiVO_4_/electrolyte interface are removed from the reaction environment through buffering. In other words, differently from what has been previously observed for PEC water oxidation,^26^ a lower buffering capacity within the electrolyte solution might correlate with an improved GOR performance. We therefore evaluated the buffering capacity of the solutions by introducing equimolar amounts of protons to the glycerol-containing solutions. For instance, 25 mmol of H_2_SO_4_ was added to 1 L of a pH 2 solution of either K_2_SO_4_ or Na_2_SO_4_ containing 0.1 M glycerol, each with an electrolyte concentration of 0.5 M. Subsequently, the pH of the resulting mixtures was measured. In a separate instance, 50 mmol of HNO_3_ or HClO_4_ was added to 1 L of a pH 2 NaNO_3_ or NaClO_4_ solution containing 0.1 M glycerol (electrolyte concentration = 0.5 M). The resultant changes in pH (ΔpH) induced by the addition of 50 mM protons are presented in Table 2. Interestingly, the NaNO_3_ and NaClO_4_ solutions exhibited considerably higher ΔpH (indicative of the lowest buffering capacity) compared to the other three solutions, whereas the KP_i_ solution demonstrated the lowest ΔpH (indicative of the highest buffering capacity). The estimated buffering capacities were ranked as follows: NaNO_3_ < NaClO_4_ < Na_2_SO_4_ < K_2_SO_4_ < KP_i_. This trend agrees well with the photocurrent trend, except for the ranking between NaClO_4_ and NaNO_3_, thus implying a potential impact of buffering action in the electrolyte on the GOR process.

**Table 2:** The impact of adding 50 mM protons to the pH of the various acidic electrolytes used in this study. All electrolytes had a concentration of 0.5 M and contained 0.1 M glycerol. The displayed values represent the averages (± standard deviation) obtained from three distinct measurements. The raw data can be found in the ESI (Table S3–S7)

	KP_i_	K_2_SO_4_	Na_2_SO_4_	NaClO_4_	NaNO_3_
Initial pH	2.00 ± 0.01	1.99 ± 0.01	1.98 ± 0.01	2.00 ± 0.01	1.99 ± 0.02
Final pH	1.93 ± 0.01	1.80 ± 0.01	1.72 ± 0.02	1.38 ± 0.03	1.19 ± 0.07
ΔpH	0.07 ± 0.01	0.19 ± 0.01	0.27 ± 0.01	0.62 ± 0.05	0.80 ± 0.09

Despite the evidence provided above, the speculative nature of these explanations is acknowledged. Further experiments beyond the scope of this study would be needed to fully reveal the mechanism behind the clearly observed performance trend. Another factor that needs to be considered is the effect of anions on the reorganization energy of glycerol in the electrolyte. As outlined in the Marcus-Gerischer theory,^27^ the charge transfer rate at a semiconductor/liquid interface depends on the alignment between the energy levels in the semiconductor (*i.e.*, conduction and valence bands) and the donor/acceptor states of the redox species in the electrolyte (in our case, glycerol). Any change in the reorganization energy, possibly induced by the presence of different anions, would change the energy gap between the donor/acceptor states of the solute. Hence, this would change the relative energy alignment at the interface and consequently modify the electron transfer kinetics. The reorganization energy of glycerol in these acidic electrolytes, which is to the best of our knowledge not yet available, needs therefore to be evaluated.

## Conclusion

In summary, the influence of various acidic electrolytes (KP_i_, K_2_SO_4_, Na_2_SO_4_, NaClO_4_, and NaNO_3_; pH = 2) on the PEC glycerol oxidation over BiVO_4_ was systematically investigated. Interestingly, we observed that BiVO_4_ exhibited the following GOR performance trend: NaClO_4_, NaNO_3_ > Na_2_SO_4_ > K_2_SO_4_ > KP_i_, with the photocurrent in NaClO_4_ ∼3-fold of that in KP_i_. Although our BiVO_4_ photoanodes exhibited the highest photocurrent in NaClO_4_, the low production rate of GOR products, due to the poor stability of BiVO_4_ in this electrolyte solution, made it less promising than NaNO_3_. NaNO_3_ emerged as the preferred electrolyte for PEC glycerol oxidation on BiVO_4_, offering superior performance in terms of photocurrent, stability, and selectivity towards value-added GOR products. Glycolaldehyde (GCAD) was identified as the most dominant GOR product in our study, achieving a selectivity higher than 50% in NaNO_3_. We attributed the observed GOR performance trend among the employed electrolytes to the various effects induced by both cations and anions on the GOR performance of BiVO_4_. First, the larger effective nuclear charge of K^+^*vs.* Na^+^ may result in reduced electrostatic adsorption of glycerol onto the BiVO_4_ surface, consistent with the lower performance in K^+^-containing electrolytes. In addition, the specific ion effects were considered as they may impact the aggregation of glycerol in the electrolyte. Our Raman spectroscopy results suggest more extensive glycerol aggregation in the presence of SO_4_^2−^ compared to NO_3_^−^, consistent with the weaker salting-out effect of NO_3_^−^ ions indicated by the Hofmeister series. Finally, we found an inverse relationship between the buffering capacity of the electrolyte and the GOR performance of BiVO_4_. The underlying mechanism is likely a combination of the factors above, and future studies should be directed towards understanding this complex process through *e.g.*, *operando* investigation of the BiVO_4_/liquid electrolyte interface during PEC GOR and an assessment of the reorganization energy of glycerol in diverse electrolytes. The general applicability of our results to other anode materials and/or whether the reaction is EC- or PEC-driven was suggested as dark EC measurements with polycrystalline Pt anodes also showed a similar GOR current density trend. Overall, our findings suggest the critical role of electrolyte selection in optimizing EC/PEC glycerol oxidation, with potential implications for other (photo)electrocatalytic reactions.

## Data availability

The data that support the findings of this study are available from the corresponding authors upon reasonable request.

## Author contributions

H. K.: investigation (sample preparation, EC, PEC, XRD, XPS, UV-vis, HPLC, solution conductivity measurements, buffering capacity measurements), data curation, visualisation, conceptualisation, writing – original draft; S. G.: investigation (HPLC), writing – review & editing; A. F. P.-T.: investigation (HPLC), writing – review & editing; C. H.: investigation (XPS), writing – review & editing; P. B.: investigation (MS), data curation, visualisation, writing – review & editing; M. T. M.: resources, writing – review & editing; R. v. d. K.: supervision, writing – review & editing; M. F.: supervision, investigation (Raman spectroscopy), data curation, visualisation, conceptualisation, writing – review & editing; F. F. A.: supervision, funding acquisition, conceptualisation, writing – review & editing.

## Conflicts of interest

There are no conflicts to declare.

## Supplementary Material

SC-015-D4SC01651C-s001
