# Supplementary material for: Electrolyte selection toward efficient photoelectrochemical glycerol oxidation on BiVO4
Source: Chem Sci. 2024 May 31;15(27):10425–35. doi: 10.1039/d4sc01651c (PMC11234828; doi:10.1039/d4sc01651c)
Supplement: SC-015-D4SC01651C-s001 [file SC-015-D4SC01651C-s001.pdf]

Electronic Supplementary Material (ESI) for Chemical Science.
This journal is © The Royal Society of Chemistry 2024

Supporting Information

Electrolyte Selection Toward Efficient Photoelectrochemical Glycerol Oxidation on $BiVO_4$

*Heejung Kong,[1] Siddharth Gupta[1, 2], Andrés F. Pérez-Torres[1], Christian Höhn[1], Peter Bogdanoff[1], Matthew T. Mayer[1, 2], Roel van de Krol[1, 3], Marco Favaro[1, *] and Fatwa F. Abdi,[1, 4, *]*

[1] Institute for Solar Fuels, Helmholtz-Zentrum Berlin fur Materialien und Energie GmbH, Hahn-Meitner-Platz 1, 14109 Berlin, Germany

[2] Institut für Chemie & Biochemie, Freie Universität Berlin, 14195 Berlin, Germany

[3] Institut für Chemie, Technische Universität Berlin, Straße des 17. Juni 124, 10623 Berlin, Germany

[4] School of Energy and Environment, City University of Hong Kong, 83 Tat Chee Avenue, Kowloon, Hong Kong S.A.R., China

[*] Authors to whom correspondence should be addressed.

M. Favaro marco.favaro@helmholtz-berlin.de

F. F. Abdi fatwa.abdi@helmholtz-berlin.de ; ffabdi@cityu.edu.hk

Table of Contents

1. Methods .... [4]

2. Figures for the Supporting Information .... [10]

- **Figure S1.** SEM image of the nanoporous $BiVO_4$ film used in our study.
- **Figure S2.** (a) XRD pattern of the $BiVO_4$ film and FTO substrate. (b) Tauc plot of the $BiVO_4$ film for the indirect bandgap estimation.
- **Figure S3.** Dark LSV curves measured in various acidic (pH = 2) electrolyte solutions (a) without glycerol and (b) with 0.1 M glycerol.
- **Figure S4.** LSV curves recorded under AM 1.5G illumination in various acidic (pH = 2) electrolyte solutions: (a) KP_i, (b) K_2SO_4, (c) Na_2SO_4, (d) $NaClO_4$, and (e) $NaNO_3$, all of which had a concentration of 0.5 M and did not contain glycerol. Three distinct $BiVO_4$ samples (Sample 1 – 3) were employed for the measurements in each solution as a reproducibility check.
- **Figure S5.** LSV curves recorded under AM 1.5G illumination in various acidic (pH = 2) electrolyte solutions: (a) KP_i, (b) K_2SO_4, (c) Na_2SO_4, (d) $NaClO_4$, and (e) $NaNO_3$, all had a concentration of 0.5 M and contained 0.1 M glycerol. Three distinct $BiVO_4$ samples (Sample 1 – 3) were employed for the measurements in each solution as a reproducibility check.
- **Figure S6.** LSV curves measured under AM 1.5G illumination in various acidic (pH = 2) electrolyte solutions containing 0.5 M of glycerol.
- **Figure S7.** (a) LSV curves of $BiVO_4$ in a pH 1 $NaNO_3$ solution containing 0.5 M glycerol. (b) CA curves recorded at 1.23 V_{RHE} for 12 hours in $NaNO_3$ solutions at pH 1 and pH 2, with an initial glycerol concentration of 0.5 M.

- **Figure S8.** X-ray photoelectron spectroscopy (XPS) Bi 4f core-level spectra of the (a) pristine sample and samples subjected to 12-hour chronoamperometry (CA) tests in (b) $NaNO_3$, (c) $NaClO_4$, (d) Na_2SO_4, and (e) K_2SO_4.
- **Figure S9.** X-ray photoelectron spectroscopy (XPS) O 1s and V 2p core-level spectra of the (a) pristine sample and samples subjected to 12-hour chronoamperometry (CA) tests in (b) $NaNO_3$, (c) $NaClO_4$, (d) Na_2SO_4, and (e) K_2SO_4.
- **Figure S10.** Calibration data for glycerol in high-performance liquid chromatography (HPLC) analysis.
- **Figure S11.** Calibration data for dihydroxyacetone (DHA) in high-performance liquid chromatography (HPLC) analysis.
- **Figure S12.** Calibration data for glycolaldehyde (GCAD), glyceraldehyde (GLAD), formic acid (FA), glyceric acid (GA), glycolic acid (GCA), and lactic acid (LA) in high-performance liquid chromatography (HPLC) analysis.
- **Figure S13.** High-performance liquid chromatography (HPLC) chromatograms for the $NaNO_3$ case.
- **Figure S14.** High-performance liquid chromatography (HPLC) chromatograms for the $NaClO_4$ case.
- **Figure S15.** High-performance liquid chromatography (HPLC) chromatograms for the Na_2SO_4 case.
- **Figure S16.** High-performance liquid chromatography (HPLC) chromatograms for the K_2SO_4 case.
- **Figure S17.** Mass spectroscopy (MS) measured in a pH 2 $NaNO_3$ solution.
- **Figure S18.** Chromatograms of aqueous ammonia (NH_3) solutions and pH 2 $NaNO_3$ solutions ($NaNO_3$ concentration = 0.5 M) obtained via high-performance liquid chromatography (HPLC).

- **Figure S19.** Full range Raman scattering spectra taken on different liquid samples, at a laser wavelength of 785 nm and a total spectral power of 450 mW. The spectrometer (Wasatch Photonics WP785ER) has an average spectral resolution of about 5 cm^{-1}. The Raman shift scale was calibrated using the Si 520 cm^{-1} mode. The spectra have been recorded at about 22 °C, using a 2 mL optical grade-quartz cuvette.
- **Figure S20.** C–O stretching band region for the spectral references, 0.5 M glycerol (a) and 0.5 $NaNO_3$ (b). The spectra have been recorded at about 22 °C, using a 2 mL optical grade-quartz cuvette, at a laser wavelength of 785 nm, and a total spectral power of 450 mW.

3. Tables for the Supporting Information .... [30]

- **Table S1.** Water oxidation photocurrent values expressed in mA cm^{-2} at 1.23 V_{RHE}, extracted from **Figure S4.**
- **Table S2.** Glycerol oxidation photocurrent values expressed in mA cm^{-2} at 1.23 V_{RHE}, extracted from **Figure S5**.
- **Table S3.** The impact of adding 50 mM protons to the pH of the 0.5 M KP_i + 0.1 M glycerol solution.
- **Table S4.** The impact of adding 50 mM protons to the pH of the 0.5 M K_2SO_4 + 0.1 M glycerol solution.
- **Table S5.** The impact of adding 50 mM protons to the pH of the 0.5 M Na_2SO_4 + 0.1 M glycerol solution.
- **Table S6.** The impact of adding 50 mM protons to the pH of the 0.5 M Na_2SO_4 + 0.1 M glycerol solution.
- **Table S7.** The impact of adding 50 mM protons to the pH of the 0.5 M $NaNO_3$ + 0.1 M glycerol solution.

1. Methods

Sample preparation

First, 0.4 M of potassium iodide (Santa Cruz Biotechnology) was completely dissolved in 50 mL of deionized water (18.2 MΩ), followed by adding 0.1 mL of nitric acid (>69.0%, Honeywell) and 0.04 M of bismuth nitrate pentahydrate (Acros Organics). The solution was stirred using a magnetic bar until the salts were fully dissolved. Second, a 20 mL ethanolic solution with 0.225 M of p-benzoquinone (Alfa Aesar) was prepared. Subsequently, the aqueous solution was slowly added to the ethanolic solution, resulting in a very dark red but clear solution. Next, BiOI nanosheet arrays were grown on FTO substrates with a sheet resistance of 7 Ω sq^{-1} (Sigma Aldrich) through electrodeposition. In the electrodeposition process, a platinum coiled wire with a diameter of 0.5 mm and an Ag/AgCl (saturated KCl) electrode (XR300, Radiometer Analytical) were employed as the counter electrode and the reference electrode, respectively. With the three electrodes immersed in the solution, a constant potential of −0.1 V vs. Ag/AgCl was applied until reaching a charge of 200 mC cm^{-2}, resulting in the formation of red-orange films. Subsequently, the BiOI films were coated with a 50 μL cm^{-2} solution of 0.2 M vanadyl acetylacetonate (Acros Organics) in dimethyl sulfoxide (VWR Life Science). The coated films were then annealed on a hot plate at 450 °C for 2 hours with a ramping rate of 5 K min^{-1} to induce the conversion into monoclinic $BiVO_4$. Finally, excess V_2O_5 layers were removed by immersing the samples into a 1 M NaOH (Sigma Aldrich) solution for 15 minutes.

Preparation of electrolyte solutions

The following chemicals were employed in the synthesis of electrolyte solutions: glycerol (>99% Sigma Aldrich), $NaNO_3$ (>99.0%, Sigma Aldrich), Na_2SO_4 (>99%, Sigma Aldrich), K_2SO_4 (>99%, Santa Cruz Biotechnology), KH_2PO_4 (>99.0%, Sigma Aldrich), K_2HPO_4 (>99.0%, Sigma Aldrich), HNO_3 (>69.0%, Honeywell), H_2SO_4 (97%, Honeywell), and H_3PO_4 (>85%,

Honeywell). Deionized water with a resistivity of 18.2 MΩ cm, produced by a water purification system (Merck Millipore), was used as a solvent. The concentration of all solutions was 0.5 M. The pH values of the solutions were carefully adjusted to 2 by adding a suitable acid (HNO_3 for $NaNO_3$, H_2SO_4 for Na_2SO_4 and K_2SO_4, or H_3PO_4 for KP_i, respectively) to prevent mixing different kinds of anions.

Electrochemical experiments

Electrochemical measurements were conducted using a potentiostat (VersaSTAT 3F, Princeton Applied Research). The same reference electrode and counter electrode used during the electrodeposition process were employed. An AM1.5G solar simulator (WACOM WXS–50S–5H Class AAA) with an irradiance of 100 mW cm^{-2} was employed as the light source. In the PEC measurements, the light was illuminated from the backside (i.e., the rear of the sample through the FTO-substrate side). The scan rate in LSV was set at 20 mV s^{-1}. Chronoamperometry was performed at 1.23 V_{RHE} under the same AM1.5G illumination. The applied potential with respect to the Ag/AgCl ($V_{Ag/AgCl}$) reference electrode was converted to the V_{RHE} scale using the following Nernst equation:

$$V_{RHE} = V_{Ag/AgCl} + 0.059 \times pH + V^0_{Ag/AgCl} \quad [1]$$

where $V^0_{Ag/AgCl}$ is the standard potential of the reference electrode (0.197 V). No iR correction was performed to present the data, considering the relatively small current (maximum current ~2 mA) and cell impedance (~10 Ω) during all electrochemical measurements.

Product analysis using high-performance liquid chromatography (HPLC)

Liquid samples, collected after 12 hours of photoelectrolysis at a constant potential of 1.23 V_{RHE} in various electrolyte solutions, were analyzed using an HPLC system (UltiMate 3000, Thermo Scientific) for quantifying glycerol oxidation reaction (GOR) products. The system was

equipped with a single column (HyperREZ XP H+, Thermo Scientific) and utilized both a wavelength-variable UV detector (UltiMate 3000, Thermo Scientific) and a refractive index (RI) detector (RefractoMax 520, Thermo Scientific). The flow rate was maintained at 0.5 mL min^{-1}, and the column temperature was held constant at 60°C. A 5 mM H_2SO_4 aqueous solution was employed as the mobile phase.

The following chemicals were used as reference GOR products: dihydroxyacetone (DHA, for synthesis, Sigma Aldrich), formic acid (FA, 98−100%, Sigma Aldrich), DL-glyceraldehyde (GLAD, >90%, Sigma Aldrich), glycolaldehyde dimer (GCAD, Sigma Aldrich), glycolic acid (GCA, 98%, Thermo Scientific), DL-glyceric acid (GA, ~2 M in water, Chem Cruz), and lactic acid (LA, 85%, Sigma Aldrich). For calibration, five aqueous solutions with varying concentrations (100−500 mM in increments of 100 mM for glycerol and 1−5 mM in increments of 1 mM for GOR products) were analyzed using the RI detector and the UV detector at 200 nm and 210 nm, as depicted in **Figure S9−S11**. We integrated the areas under their peaks to establish a linear relationship between the concentration and the peak area, also shown in **Figure S9−S11**. Selectivity (*S*) towards product *i* (e.g., S_{GCAD}) was calculated using the following formula:

$$S_i\ (\%) = 100 \times mol_i/mol_{total} \quad [2]$$

where mol_i represents the amount of product *i* in moles, and mol_{total} represents the total amount of all products, also in moles. For example, since GCAD, GLAD, DHA, and FA were the only products produced in our case, mol_{total} was calculated using the following formula:

$$mol_{total} = mol_{GCAD} + mol_{GLAD} + mol_{DHA} + mol_{FA} \quad [3]$$

Faradaic efficiency (FE) was calculated using the following formula:

$$FE\ (\%) = 100 \times Q_{GOR}/Q_{total} \quad [4]$$

where Q_{total} is the total charge passed during the photoelectrolysis, and Q_{GOR} represents the charge used to oxidize glycerol. Q_{GOR} can be calculated using the following formula:

$$Q_{GOR} = \sum_i^{all} mol_i \times q_i \quad [5]$$

where q_i represents the molar charge (C mol^{-1}) that is used to produce product i (e.g., q_{GCAD}). The value of q_i varies depending on the product (q_{GCAD} = 4/3, q_{GLAD} = q_{DHA} = 2, and q_{FA} = 8/3) based on the stoichiometry of the respective reactions:

$$C_3H_8O_3 \rightarrow C_3H_6O_3 + 2H^+ + 2e^- \quad (C_3H_6O_3 = \text{GLAD and DHA})$$

$$2C_3H_8O_3 \rightarrow 3C_2H_4O_2 + 4H^+ + 4e^- \quad (C_2H_4O_2 = \text{GCAD})$$

$$C_3H_8O_3 + 3H_2O \rightarrow 3CH_2O_2 + 8H^+ + 8e^- \quad (CH_2O_2 = \text{FA})$$

Characterizations

X-ray diffraction (XRD) was conducted using an X-ray diffractometer (X'Pert, PANalytical). A Cu Kα radiation with a wavelength of 1.5406 Å was employed, and the incident angle of the X-ray was set to 2°. X-ray photoelectron spectroscopy (XPS) analyses were performed using a monochromatic Al Kα X-ray source (Focus 500, Specs), with a photon energy of 1486.84 eV, and an electron analyzer (Phoibos 100, Specs). Binding energy (BE) calibration was performed by referencing the peak position of Au $4f_{7/2}$ at 84.0 eV. Scanning electron microscopy (SEM) was carried out using a GeminiSEM 360 instrument (ZEISS). UV-Vis spectroscopy was performed using a Lambda 950 spectrophotometer (PerkinElmer). The electrical conductivity of the electrolyte solutions was measured using a Crison Basic 30 conductivity meter. Raman measurements were performed using a customized fiber-coupled system (Wasatch Photonics WP785ER), operating at NIR with a laser wavelength of 785 nm, and at a total spectral power tunable between 5 and 450 mW. The spectrometer has an average spectral resolution of about 5 cm^{-1} within the probed energy range (260 cm^{-1}–3600 cm^{-1}). The Raman shift scale was

calibrated using the Si 520 cm^{-1} mode, while the total spectral power at the focal length (about 11 mm from the objective lens) was calibrated using a Si photodiode (Thorlabs PM16-121). The spectra of the pristine liquid samples were recorded at about 22 °C, using a 2 mL optical grade-quartz cuvette. The temperature was measured in close proximity to the sample and logged throughout the measurements using a local temperature probe (Thorlabs TSP01-TH). The integration time for all the measurements reported in this work was set to 2000 ms. To obtain a satisfactory statistics on the collected spectra, multiple spectra (about 25) were collected and averaged.

2. Figures for the Supporting Information

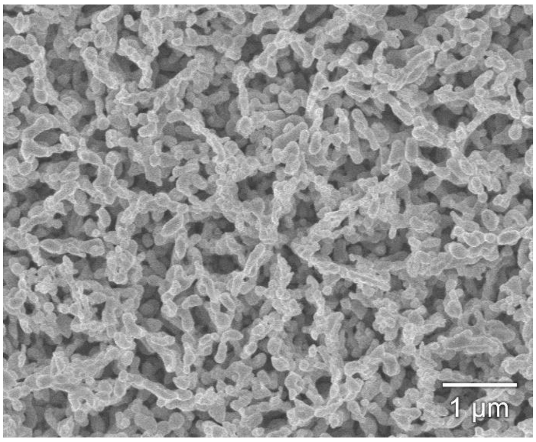

Figure S1. SEM image of the nanoporous $BiVO_4$ film used in our study.

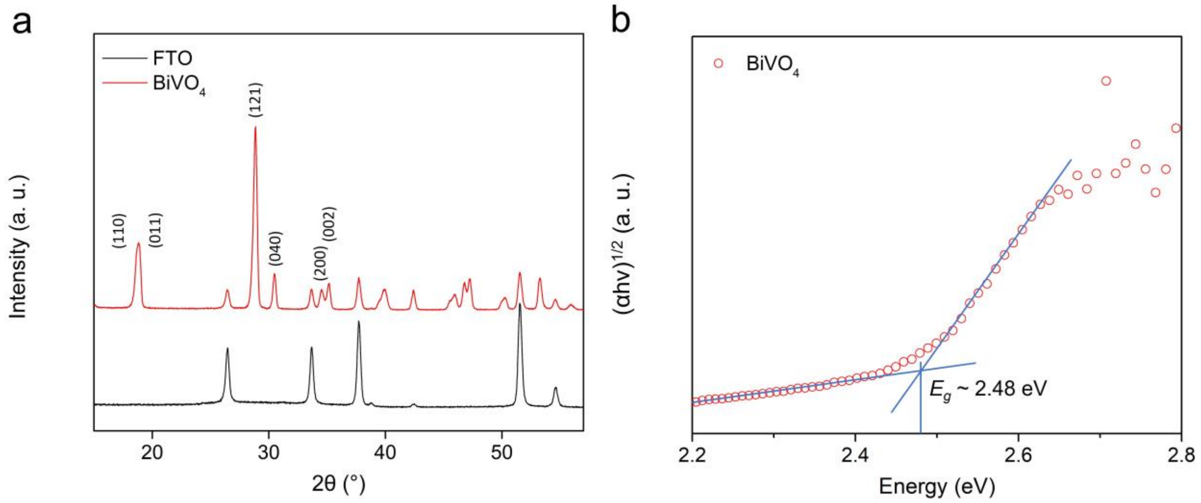

Figure S2. (a) XRD pattern of the $BiVO_4$ film and FTO substrate. **(b)** Tauc plot of the $BiVO_4$ film for the indirect bandgap estimation.

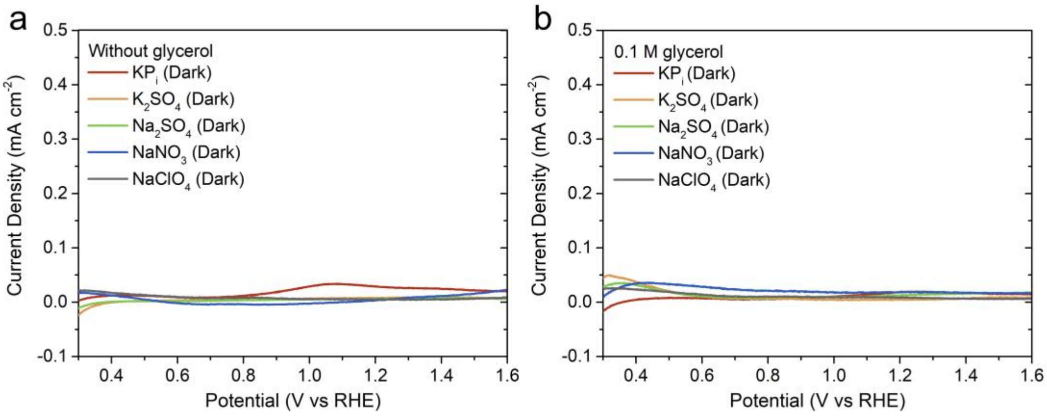

Figure S3. Dark LSV curves measured in various acidic (pH = 2) electrolyte solutions **(a)** without glycerol and **(b)** with 0.1 M glycerol.

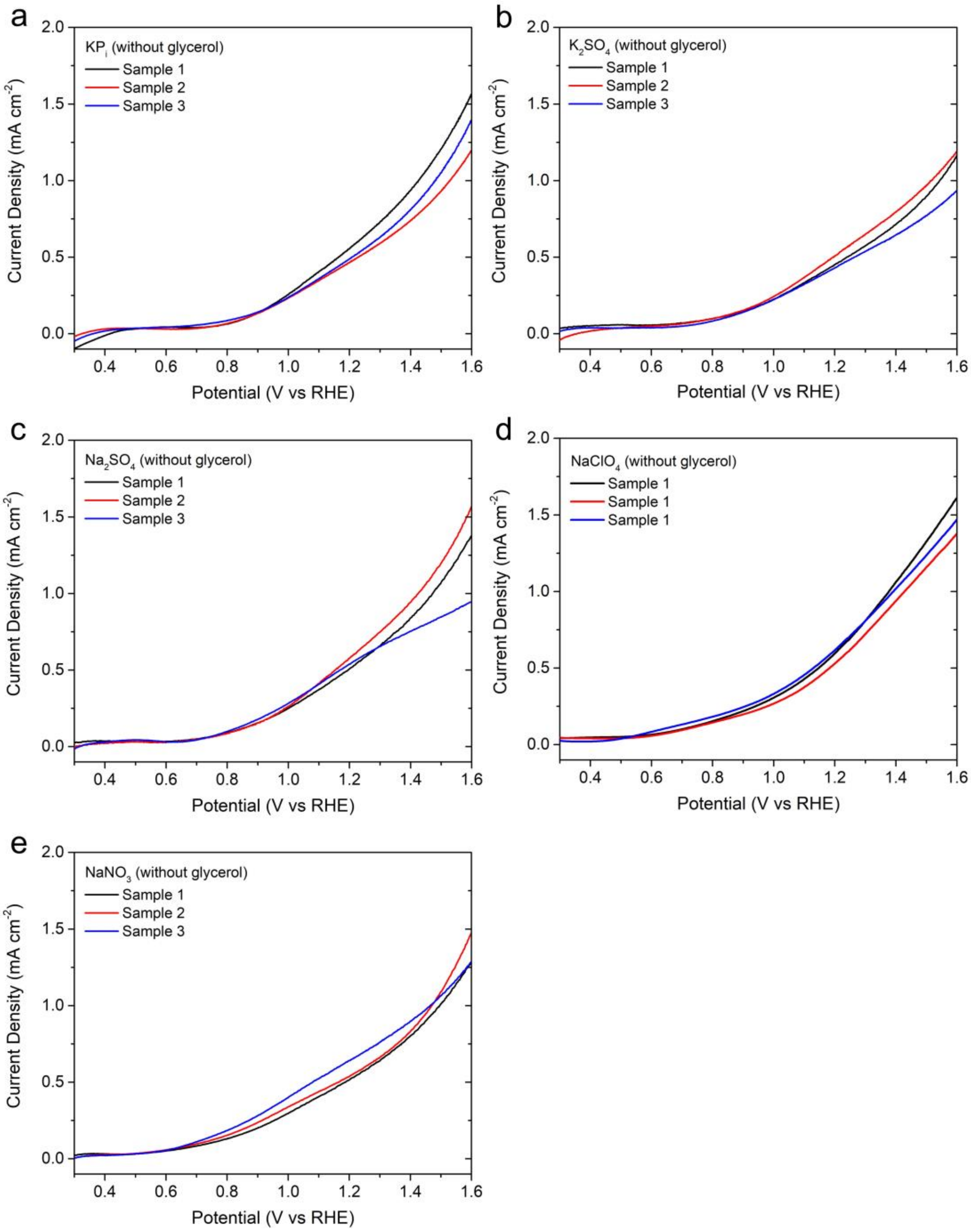

Figure S4. LSV curves recorded under AM 1.5G illumination in various acidic (pH = 2) electrolyte solutions: **(a)** KP_i, **(b)** K_2SO_4, **(c)** Na_2SO_4, **(d)** $NaClO_4$, and **(e)** $NaNO_3$, all of which had a concentration of 0.5 M and did not contain glycerol. Three distinct $BiVO_4$ samples (Sample 1 – 3) were employed for the measurements in each solution as a reproducibility check.

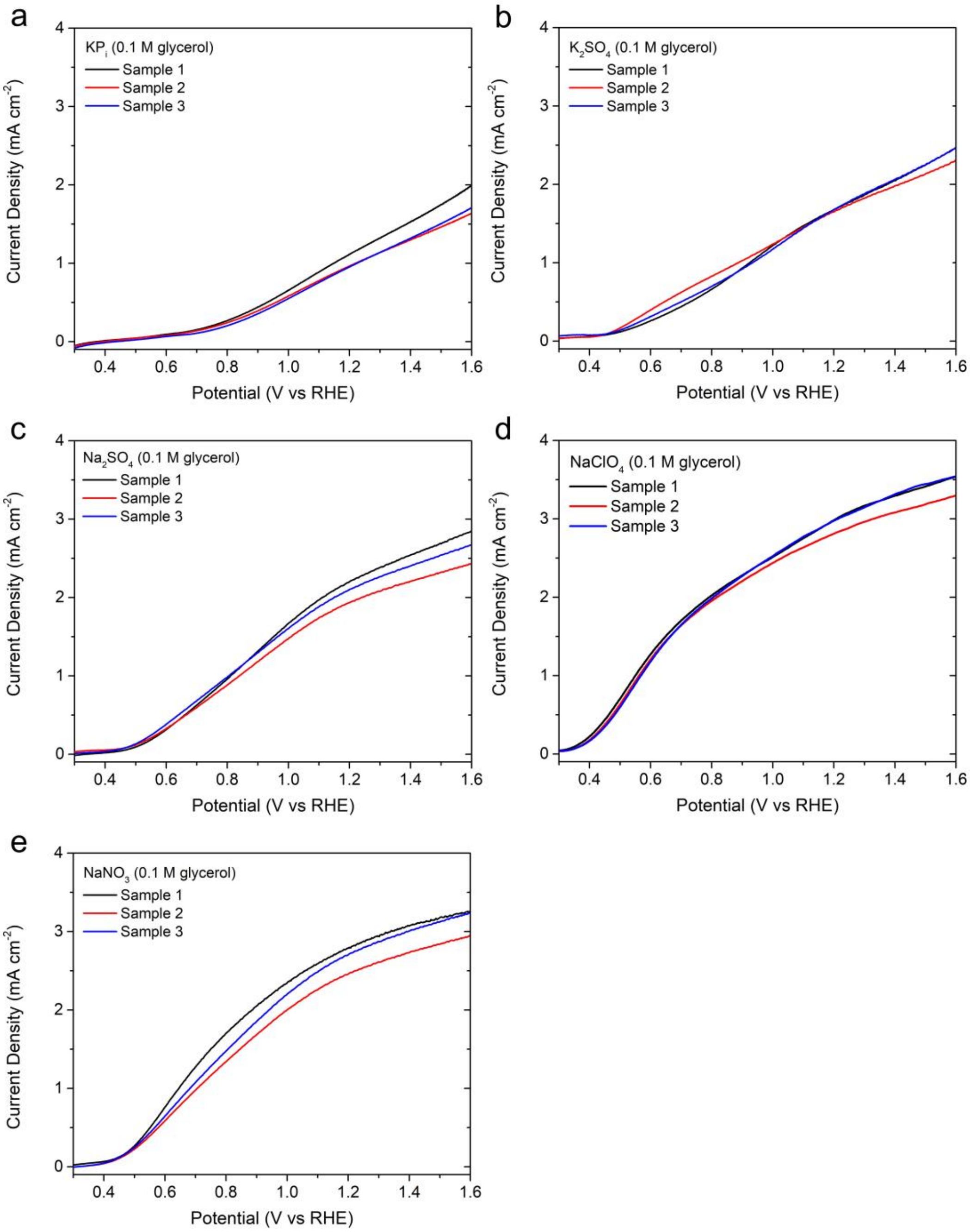

Figure S5. LSV curves recorded under AM 1.5G illumination in various acidic (pH = 2) electrolyte solutions: **(a)** KP_i, **(b)** K_2SO_4, **(c)** Na_2SO_4, **(d)** $NaClO_4$, and **(e)** $NaNO_3$, all had a concentration of 0.5 M *and* contained 0.1 M glycerol. Three distinct $BiVO_4$ samples (Sample 1 – 3) were employed for the measurements in each solution as a reproducibility check.

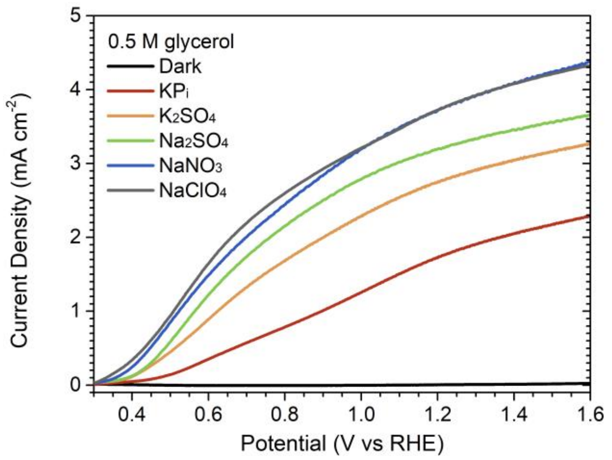

Figure S6. LSV curves measured under AM 1.5G illumination in various acidic (pH = 2) electrolyte solutions containing 0.5 M of glycerol.

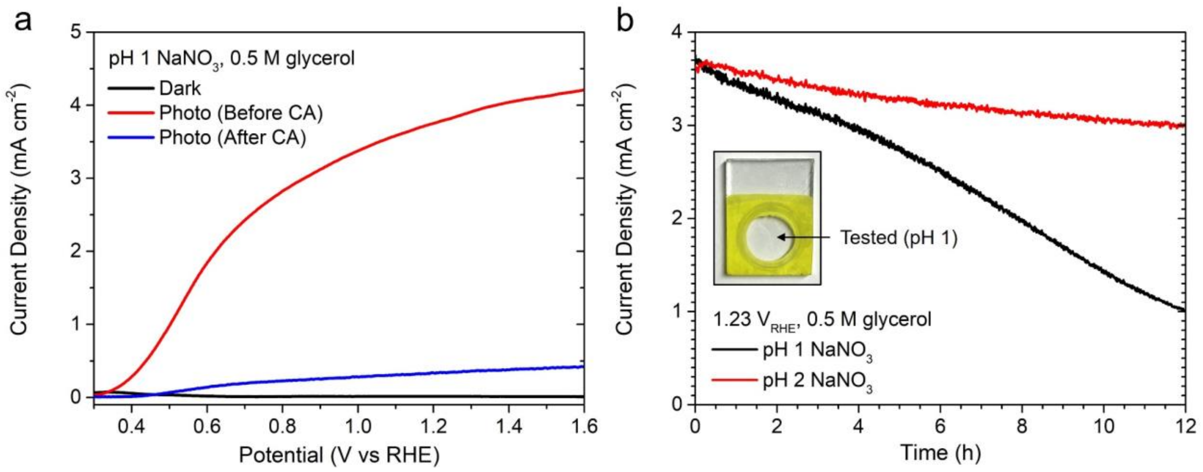

Figure S7. (a) Linear sweep voltammetry (LSV) curves of $BiVO_4$ in a pH 1 $NaNO_3$ solution containing 0.5 M glycerol ($NaNO_3$ concentration = 0.5 M). The red and blue curves represent the LSV measurements taken before (red) and after (blue) the chronoamperometry (CA) measurements shown in **(b)**. **(b)** CA curves recorded at 1.23 V_{RHE} for 12 hours in $NaNO_3$ solutions at pH 1 (black) and pH 2 (red), with an initial glycerol concentration of 0.5 M. The inset in **(b)** shows the digital photograph of the sample taken after the 12-hour CA in the pH 1 $NaNO_3$ solution.

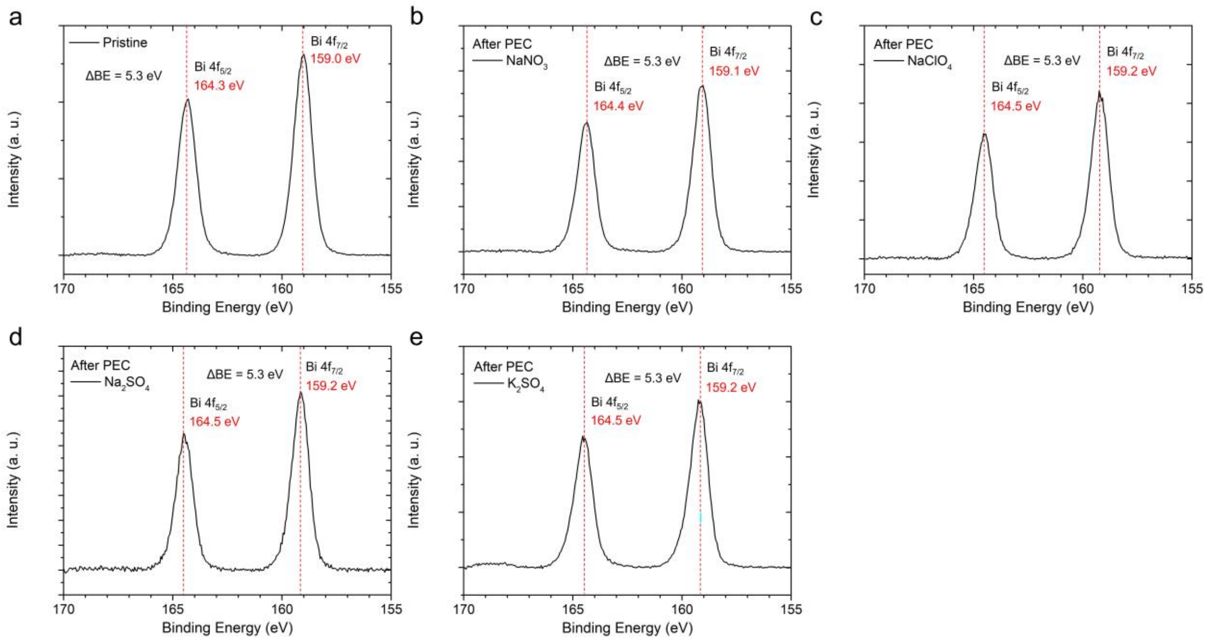

Figure S8. X-ray photoelectron spectroscopy (XPS) Bi 4f core-level spectra of the **(a)** pristine sample and samples subjected to 12-hour chronoamperometry (CA) tests in **(b)** $NaNO_3$, **(c)** $NaClO_4$, **(d)** Na_2SO_4, and **(e)** K_2SO_4.

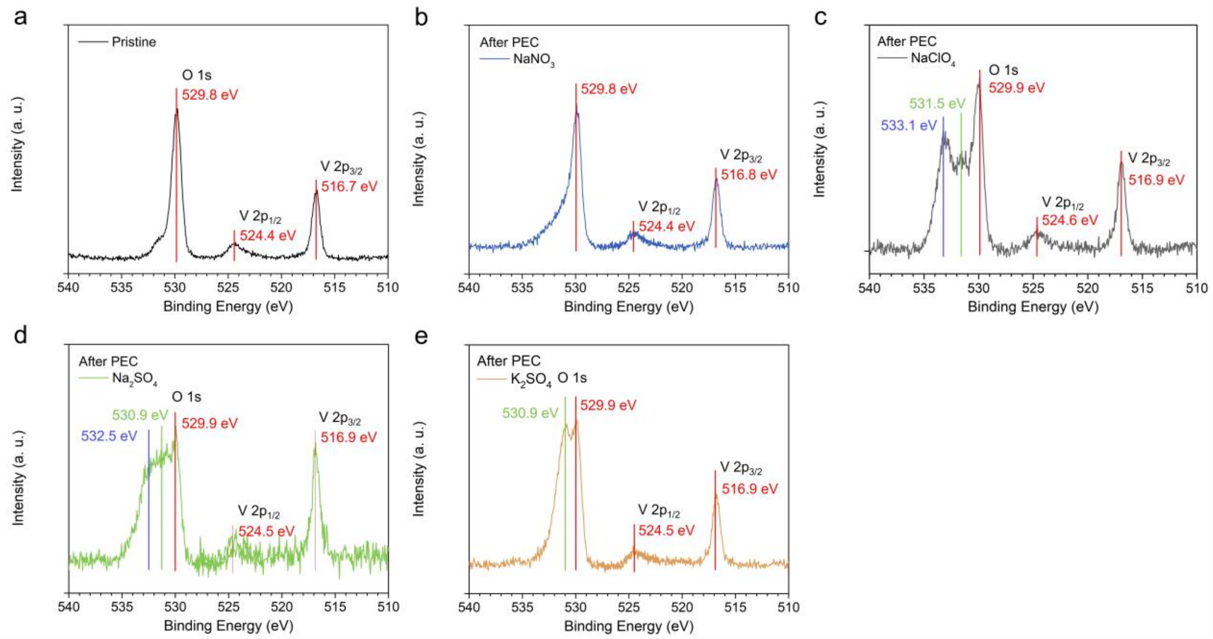

Figure S9. X-ray photoelectron spectroscopy (XPS) O 1s and V 2p core-level spectra of the **(a)** pristine sample and samples subjected to 12-hour chronoamperometry (CA) tests in **(b)** $NaNO_3$, **(c)** $NaClO_4$, **(d)** Na_2SO_4, and **(e)** K_2SO_4.

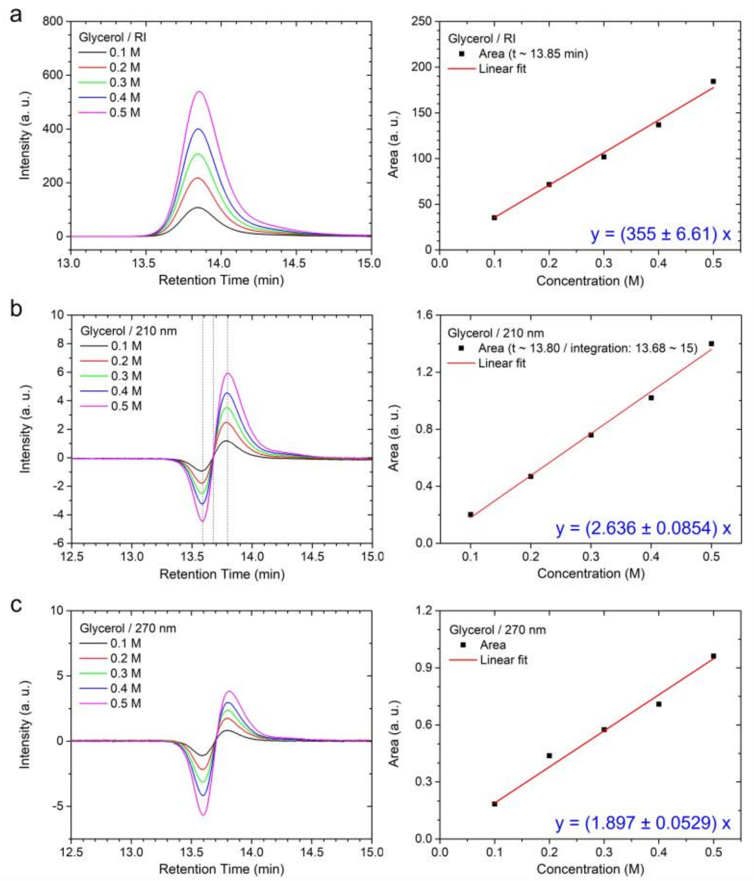

Figure S10. Calibration data for glycerol used in high-performance liquid chromatography (HPLC) analysis using **(a)** a refractive index (RI) detector and UV detectors at **(b)** 210 nm and **(c)** 270 nm wavelengths. Each plot consists of two panels: the left panel displays the chromatogram, while the right panel illustrates the linear relationship between the peak area and the concentration of glycerol.

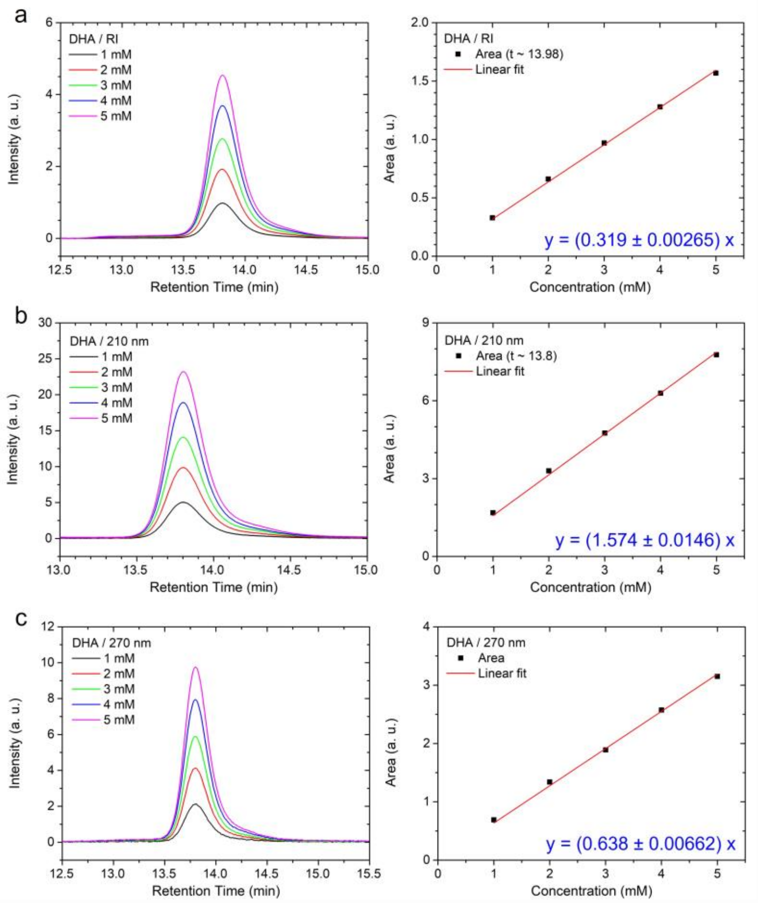

Figure S11. Calibration data for dihydroxyacetone (DHA) used in high-performance liquid chromatography (HPLC) analysis using **(a)** a refractive index (RI) detector and UV detectors at **(b)** 210 nm and **(c)** 270 nm wavelengths. Each plot consists of two panels: the left panel displays the chromatogram, while the right panel illustrates the linear relationship between the peak area and the concentration of DHA.

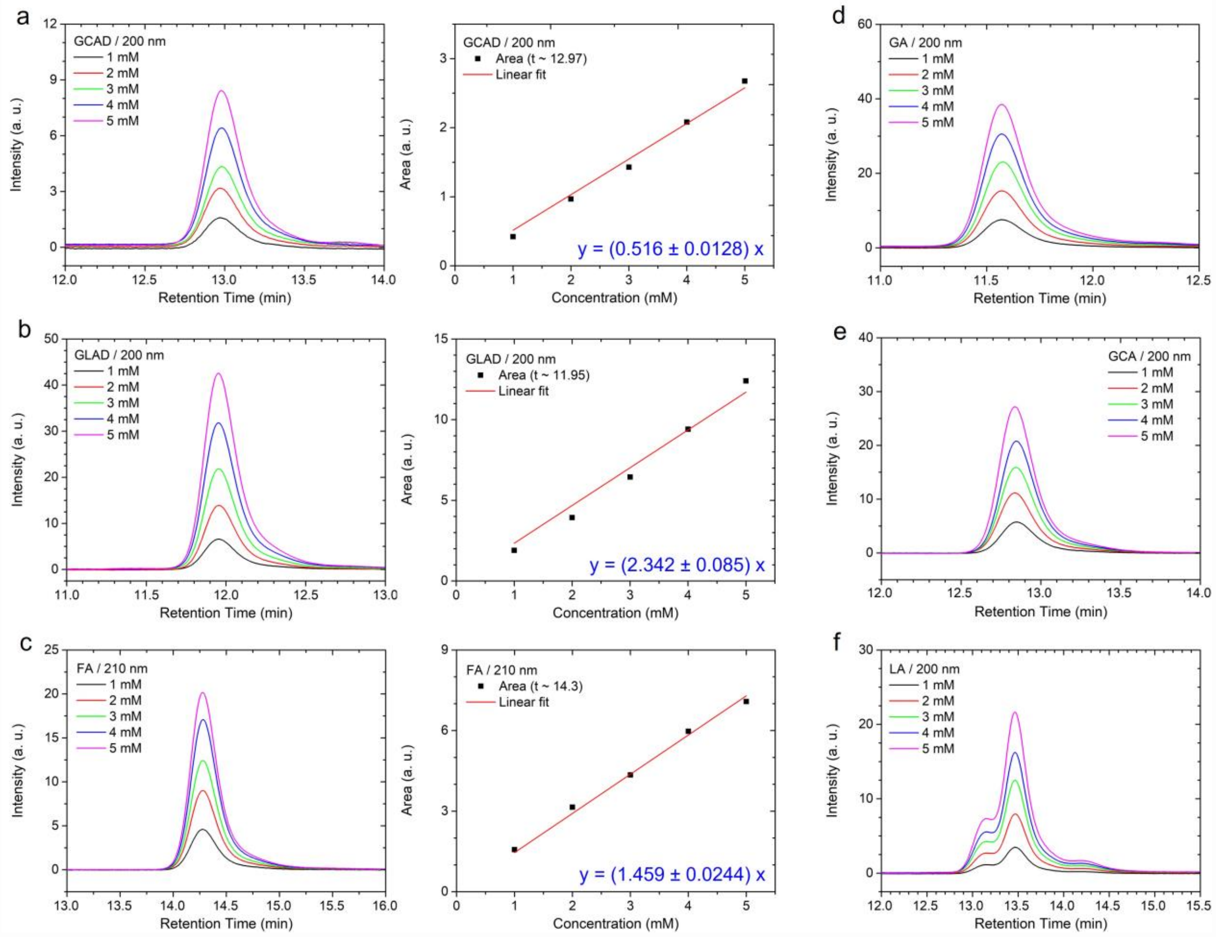

Figure S12. Calibration data for **(a)** glycolaldehyde (GCAD), **(b)** glyceraldehyde (GLAD), **(c)** formic acid (FA), **(d)** glyceric acid (GA), **(e)** glycolic acid (GCA), and **(f)** lactic acid (LA) used in high-performance liquid chromatography (HPLC) analysis. Except for FA, which was analyzed using a UV detector at 210 nm, the other products were analyzed using a UV detector at 200 nm. For **(a)** to **(c)**, the left panel displays the chromatogram, while the right panel illustrates the linear relationship between the peak area and each product's concentration. For GA, GCA, and LA, a peak area-concentration plot is not provided, as these chemicals were not detected in our experiments (refer to **Figure S13–S16**).

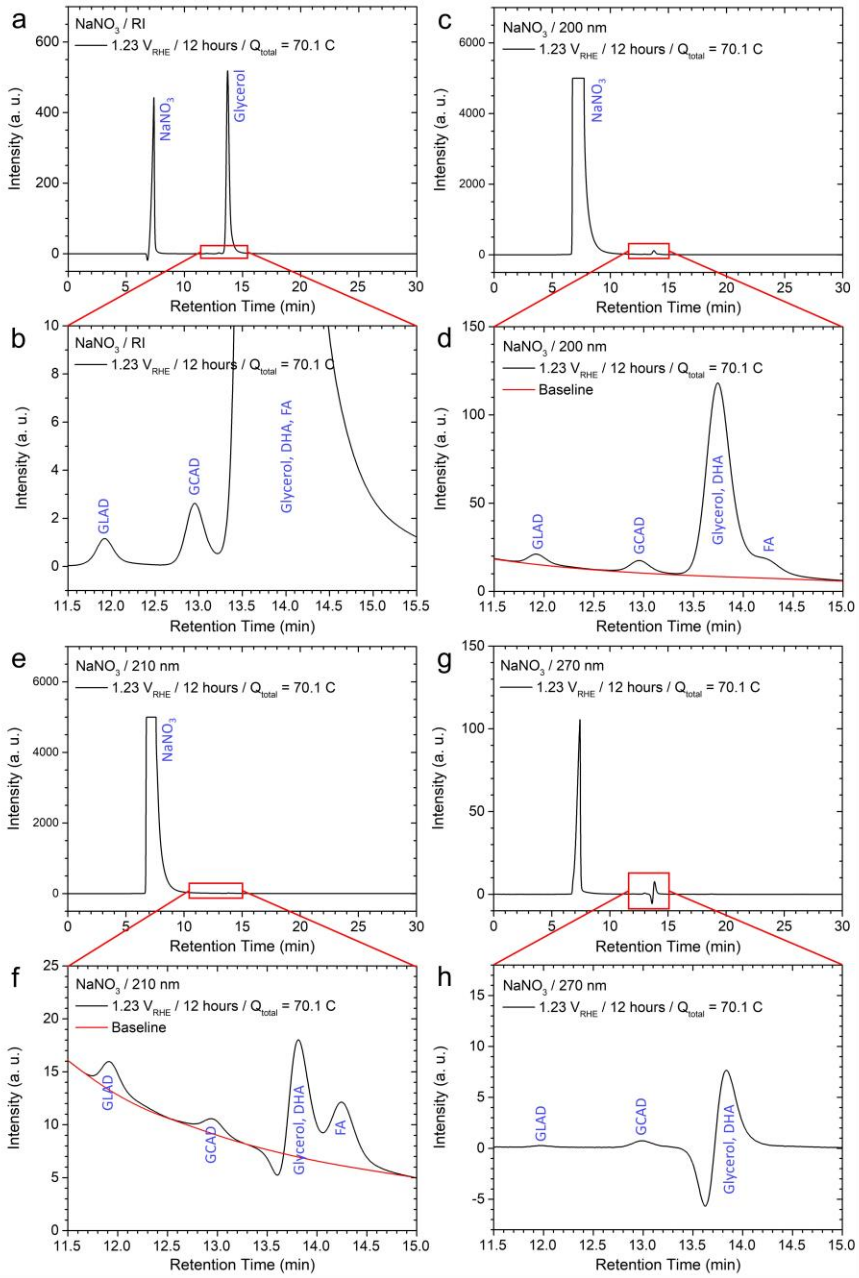

Figure S13. Product analysis using high-performance liquid chromatography (HPLC) in the $NaNO_3$ case. Chronoamperometry was performed in a pH 2 $NaNO_3$ (0.5 M) solution containing 0.5 M glycerol at a constant potential of 1.23 V_{RHE} for 12 hours, after which the solution was collected and analyzed. Chromatograms obtained using **(a, b)** a refractive index (RI) detector, **(c, d)** a UV detector at 200 nm, **(e, f)** a UV detector at 210 nm, and **(g, h)** a UV detector at 270 nm are shown. The lower row plots **(b)**, **(d)**, **(f)**, and **(h)** display magnified chromatograms of the regions highlighted by red rectangles in the upper row plots.

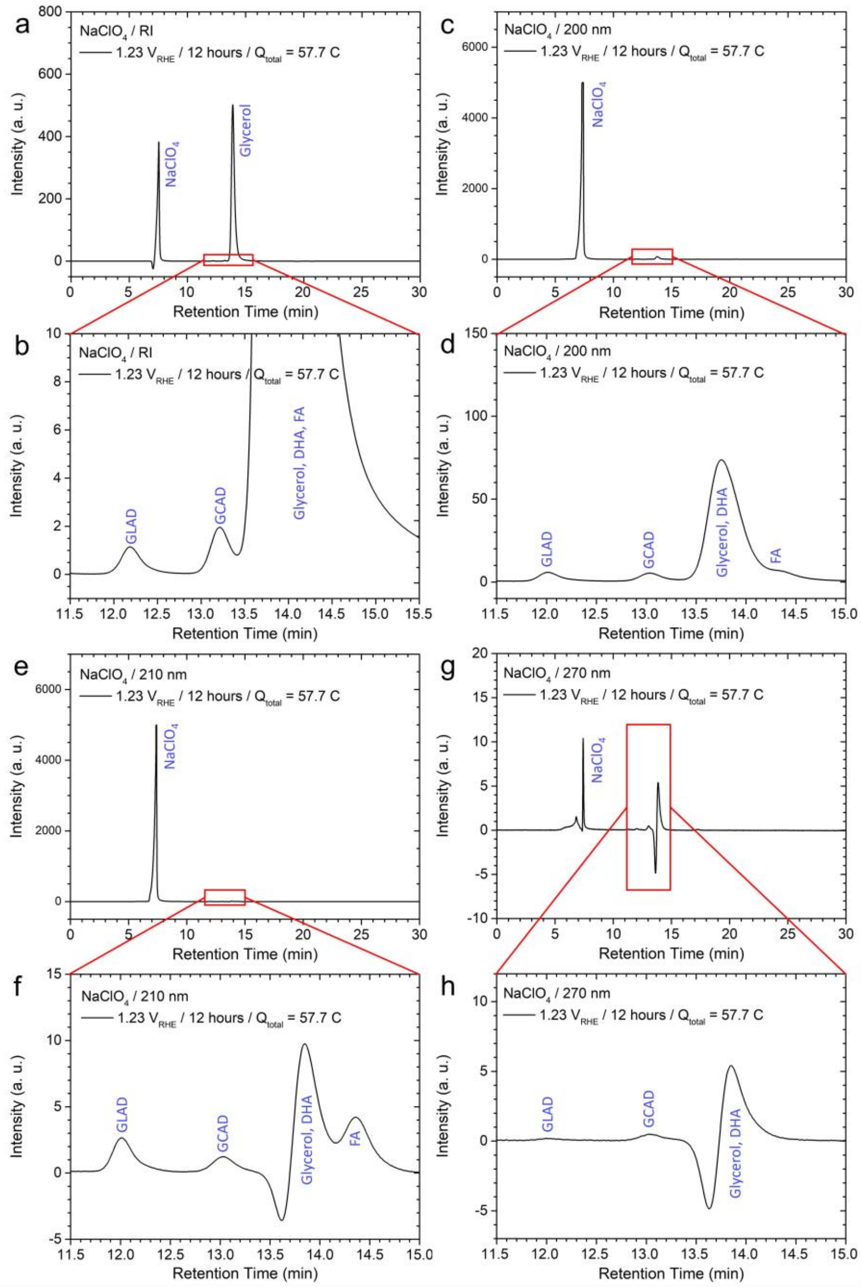

Figure S14. Product analysis using high-performance liquid chromatography (HPLC) in the $NaClO_4$ case. Chronoamperometry was performed in a pH 2 $NaClO_4$ (0.5 M) solution containing 0.5 M glycerol at a constant potential of 1.23 V_{RHE} for 12 hours, after which the solution was collected and analyzed. Chromatograms obtained using **(a, b)** a refractive index (RI) detector, **(c, d)** a UV detector at 200 nm, **(e, f)** a UV detector at 210 nm, and **(g, h)** a UV detector at 270 nm are shown. The lower row plots **(b)**, **(d)**, **(f)**, and **(h)** display magnified chromatograms of the regions highlighted by red rectangles in the upper row plots.

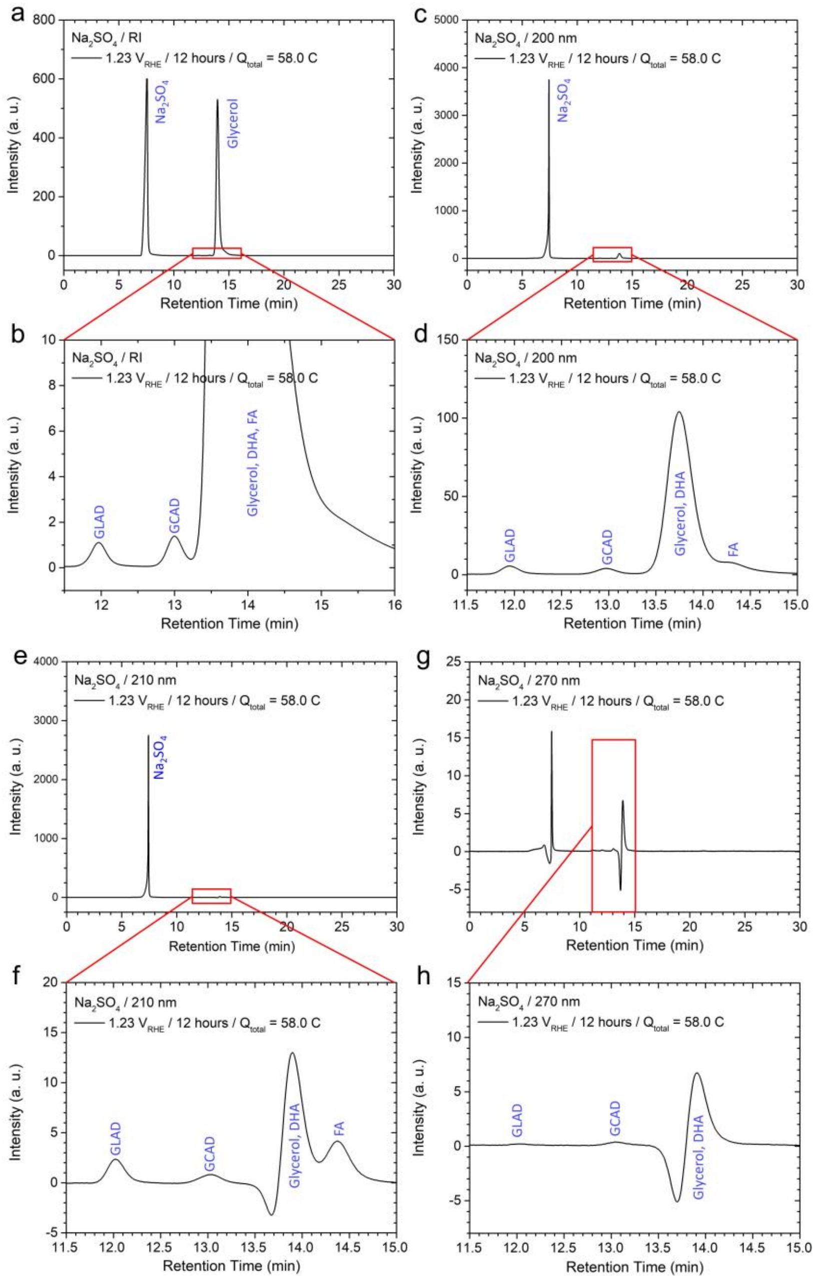

Figure S15. Product analysis using high-performance liquid chromatography (HPLC) in the Na_2SO_4 case. Chronoamperometry was performed in a pH 2 Na_2SO_4 (0.5 M) solution containing 0.5 M glycerol at a constant potential of 1.23 V_{RHE} for 12 hours, after which the solution was collected and analyzed. Chromatograms obtained using **(a, b)** a refractive index (RI) detector, **(c, d)** a UV detector at 200 nm, **(e, f)** a UV detector at 210 nm, and **(g, h)** a UV detector at 270 nm are shown. The lower row plots **(b)**, **(d)**, **(f)**, and **(h)** display magnified chromatograms of the regions highlighted by red rectangles in the upper row plots.

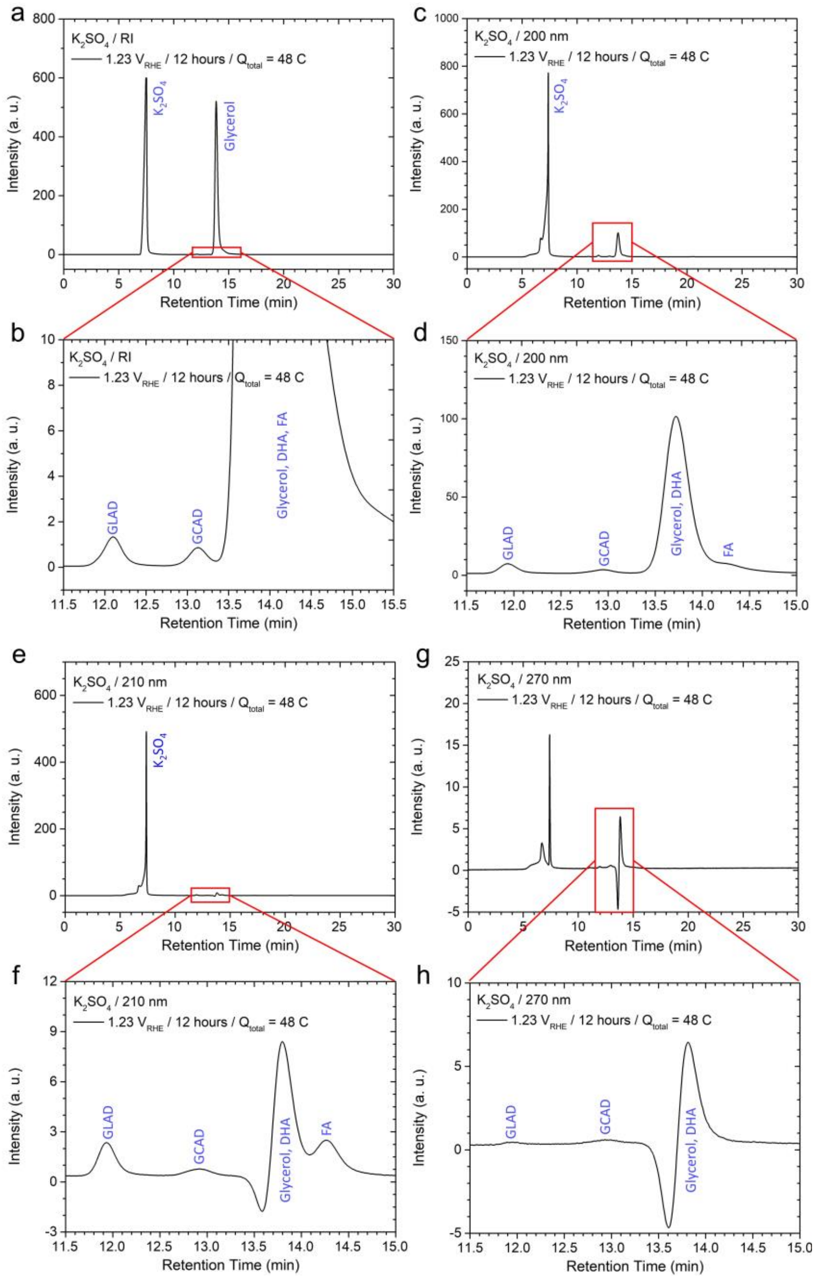

Figure S16. Product analysis using high-performance liquid chromatography (HPLC) in the K_2SO_4 case. Chronoamperometry was performed in a pH 2 K_2SO_4 (0.5 M) solution containing 0.5 M glycerol at a constant potential of 1.23 V_{RHE} for 12 hours, after which the solution was collected and analyzed. Chromatograms obtained using **(a, b)** a refractive index (RI) detector, **(c, d)** a UV detector at 200 nm, **(e, f)** a UV detector at 210 nm, and **(g, h)** a UV detector at 270 nm are shown. The lower row plots **(b)**, **(d)**, **(f)** and **(h)** display magnified chromatograms of the regions highlighted by red rectangles in the upper row plots.

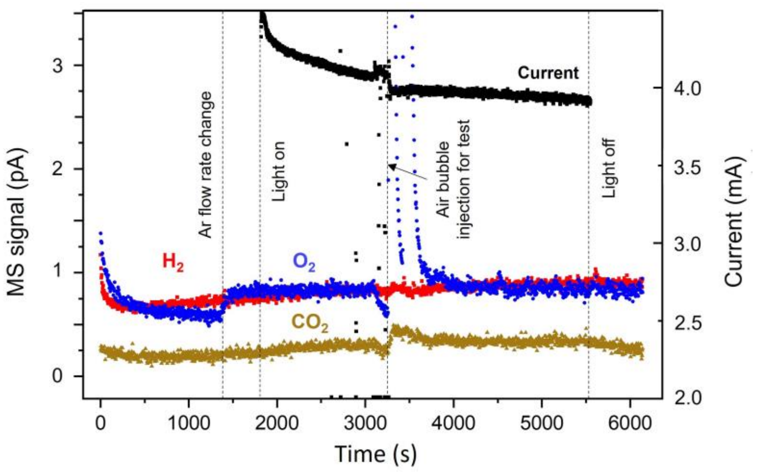

Figure S17. Mass spectroscopy (MS) result obtained from PEC measurement with $BiVO_4$ under AM1.5 illumination at a constant potential of 1.23 V_{RHE} in pH 2 $NaNO_3$ solution containing 0.5 M glycerol. During the measurement, the anolyte was separated using a Nafion membrane. The outlet of the anolyte was connected to a micro-capillary tube that was further connected to a mass spectrometer (HPR-40, HIDEN Analytical).

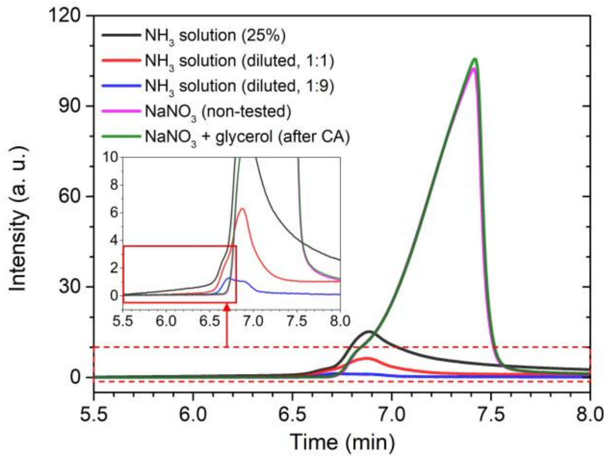

Figure S18. Chromatograms of aqueous ammonia (NH_3) solutions and pH 2 $NaNO_3$ solutions ($NaNO_3$ concentration = 0.5 M) obtained via high-performance liquid chromatography (HPLC). NH_3 solutions were prepared by diluting a 25% assay aqueous NH_3 solution with deionized water at ratios of 1:1 (red curve) and 1:9 (blue curve); for instance, in the 1:9 dilution, 10 mL of ammonia solution was mixed with 90 mL of deionized water. Signals that peak at 6.9 min with onset of < 6.5 min are attributed to NH_3. The magenta curve represents the chromatogram of the pH 2 $NaNO_3$ solution, and the olive curve represents the chromatogram of the $NaNO_3$ solution, initially containing 0.5 M glycerol, obtained after the photoelectrochemical (PEC) chronoamperometry measurement conducted at 1.23 V_{RHE} for 12 hours. If nitrate reduction reaction (NRR) occurs at the cathode during our experiments, signals of NH_3 are therefore expected in the chromatogram of $NaNO_3$ solutions after the PEC measurements. The fact that there is no feature observed until ~6.7 min (the onset of $NaNO_3$ signal that peaks at ~7.2 min) suggests that NRR cannot be detected in our experiments.

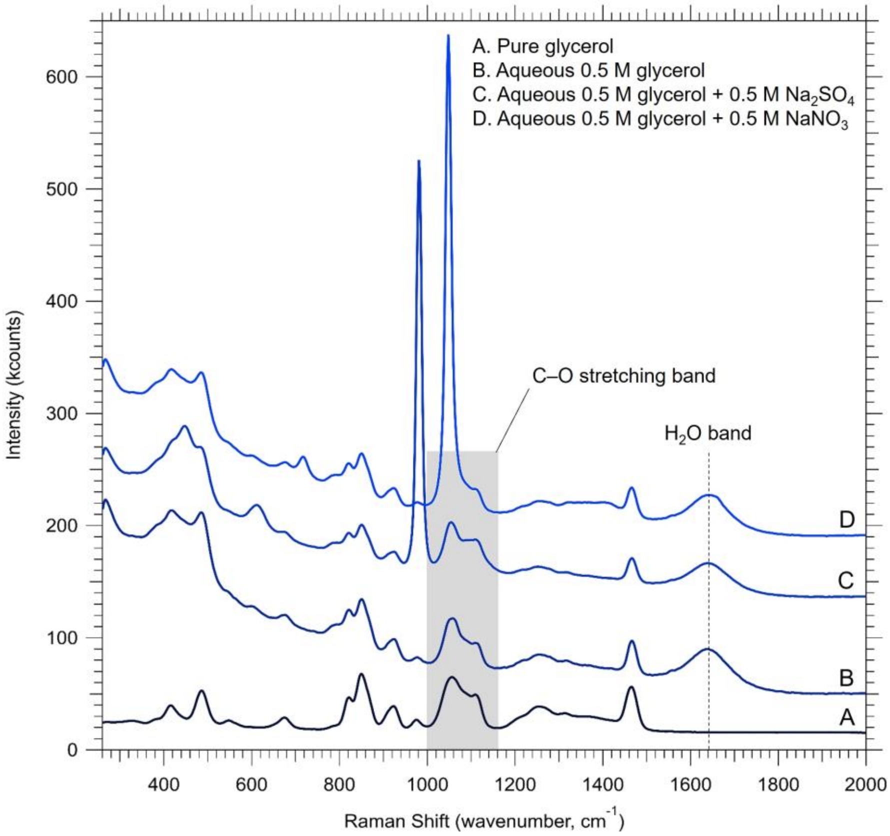

Figure S19. Full range Raman scattering spectra taken on different liquid samples, at a laser wavelength of 785 nm and a total spectral power of 450 mW. The spectrometer (Wasatch Photonics WP785ER) has an average spectral resolution of about 5 cm^{-1}. The Raman shift scale was calibrated using the Si 520 cm^{-1} mode. The spectra have been recorded at about 22 °C, using a 2 mL optical grade-quartz cuvette.

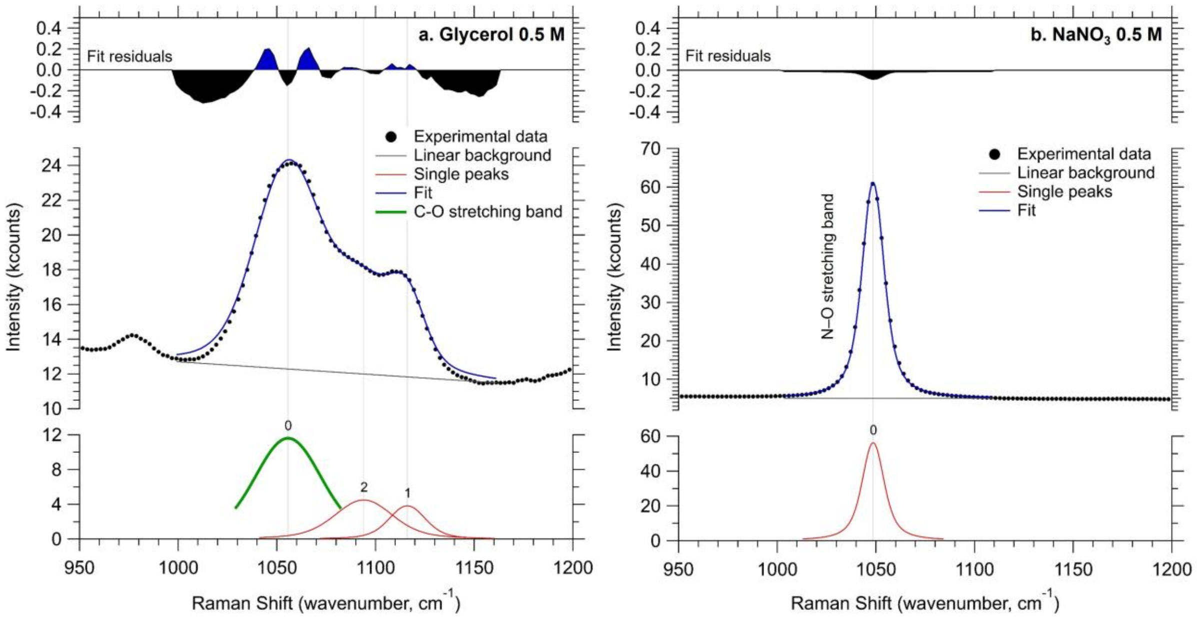

Figure S20. C–O stretching band region for the spectral references, 0.5 M glycerol (a) and 0.5 $NaNO_3$ (b). The spectra have been recorded at about 22 °C, using a 2 mL optical grade-quartz cuvette, at a laser wavelength of 785 nm, and a total spectral power of 450 mW.

3. Tables for the Supporting Information

Table S1. Water oxidation photocurrent values expressed in mA cm^{-2} at 1.23 V_{RHE}, extracted from **Figure S4**.

Without glycerol	KP_i	K_2SO_4	Na_2SO_4	$NaNO_3$	$NaClO_4$
Sample 1	0.60	0.49	0.55	0.55	0.63
Sample 2	0.50	0.55	0.63	0.57	0.59
Sample 3	0.53	0.46	0.58	0.68	0.68
Average (± standard deviation)	0.55 ± 0.04	0.50 ± 0.04	0.59 ± 0.03	0.60 ± 0.05	0.64 ± 0.04

Table S2. Glycerol oxidation photocurrent values expressed in mA cm^{-2} at 1.23 V_{RHE}, extracted from **Figure S5**.

0.1 M glycerol	KP_i	K_2SO_4	Na_2SO_4	$NaClO_4$	$NaNO_3$
Sample 1	1.18	1.74	2.26	2.82	2.85
Sample 2	1.02	1.71	1.98	3.09	2.51
Sample 3	1.01	1.74	2.15	3.00	2.76
Average (± standard deviation)	1.07 ± 0.08	1.73 ± 0.02	2.13 ± 0.11	2.97 ± 0.11	2.70 ± 0.14

Table S3. The impact of adding 50 mM protons to the pH of the 0.5 M KP_i + 0.1 M glycerol solution.

0.5 M KP_i + 0.1 M glycerol	Experiment 1	Experiment 2	Experiment 2	Average
Initial pH	2.01	2.00	1.99	2.00 ± 0.01
Final pH	1.94	1.93	1.93	1.93 ± 0.01
ΔpH	0.07	0.07	0.06	0.07 ± 0.01

Table S4. The impact of adding 50 mM protons to the pH of the 0.5 M K_2SO_4 + 0.1 M glycerol solution.

0.5 M K_2SO_4 + 0.1 M glycerol	Experiment 1	Experiment 2	Experiment 2	Average
Initial pH	1.98	1.99	2.01	1.99 ± 0.01
Final pH	1.80	1.79	1.81	1.80 ± 0.01
ΔpH	0.18	0.20	0.20	0.19 ± 0.01

Table S5. The impact of adding 50 mM protons to the pH of the 0.5 M Na_2SO_4 + 0.1 M glycerol solution.

0.5 M Na_2SO_4 + 0.1 M glycerol	Experiment 1	Experiment 2	Experiment 2	Average
Initial pH	1.98	1.97	2.00	1.98 ± 0.01
Final pH	1.70	1.70	1.75	1.72 ± 0.02
ΔpH	0.28	0.27	0.25	0.27 ± 0.01

Table S6. The impact of adding 50 mM protons to the pH of the 0.5 M $NaClO_4$ + 0.1 M glycerol solution.

0.5 M $NaClO_4$ + 0.1 M glycerol	Experiment 1	Experiment 2	Experiment 2	Average
Initial pH	1.98	2.01	1.99	2.00 ± 0.01
Final pH	1.39	1.34	1.40	1.38 ± 0.03
ΔpH	0.59	0.67	0.59	0.62 ± 0.05

Table S7. The impact of adding 50 mM protons to the pH of the 0.5 M $NaNO_3$ + 0.1 M glycerol solution.

0.5 M $NaNO_3$ + 0.1 M glycerol	Experiment 1	Experiment 2	Experiment 2	Average
Initial pH	2.02	1.98	1.97	1.99 ± 0.02
Final pH	1.12	1.18	1.28	1.19 ± 0.07
ΔpH	0.90	0.80	0.69	0.80 ± 0.09
